# COSMO-Onset: A Neurally-Inspired Computational Model of Spoken Word Recognition, Combining Top-Down Prediction and Bottom-Up Detection of Syllabic Onsets

**DOI:** 10.3389/fnsys.2021.653975

**Published:** 2021-08-04

**Authors:** Mamady Nabé, Jean-Luc Schwartz, Julien Diard

**Affiliations:** ^1^Université Grenoble Alpes, CNRS, GIPSA-Lab, Grenoble, France; ^2^Université Grenoble Alpes, CNRS, Laboratoire de Psychologie et NeuroCognition, Grenoble, France

**Keywords:** Bayesian modeling, speech perception, neural oscillations, spoken word recognition, top-down prediction, bottom-up event detection, syllabic parsing

## Abstract

Recent neurocognitive models commonly consider speech perception as a hierarchy of processes, each corresponding to specific temporal scales of collective oscillatory processes in the cortex: 30–80 Hz gamma oscillations in charge of phonetic analysis, 4–9 Hz theta oscillations in charge of syllabic segmentation, 1–2 Hz delta oscillations processing prosodic/syntactic units and the 15–20 Hz beta channel possibly involved in top-down predictions. Several recent neuro-computational models thus feature theta oscillations, driven by the speech acoustic envelope, to achieve syllabic parsing before lexical access. However, it is unlikely that such syllabic parsing, performed in a purely bottom-up manner from envelope variations, would be totally efficient in all situations, especially in adverse sensory conditions. We present a new probabilistic model of spoken word recognition, called COSMO-Onset, in which syllabic parsing relies on fusion between top-down, lexical prediction of onset events and bottom-up onset detection from the acoustic envelope. We report preliminary simulations, analyzing how the model performs syllabic parsing and phone, syllable and word recognition. We show that, while purely bottom-up onset detection is sufficient for word recognition in nominal conditions, top-down prediction of syllabic onset events allows overcoming challenging adverse conditions, such as when the acoustic envelope is degraded, leading either to spurious or missing onset events in the sensory signal. This provides a proposal for a possible computational functional role of top-down, predictive processes during speech recognition, consistent with recent models of neuronal oscillatory processes.

## 1. Introduction

### 1.1. Neural Oscillations and Multi-Scale Speech Analysis

Speech processing is classically conceived as a hierarchical process which can be broken down into several processing steps, from the low-level extraction of phonetic and prosodic cues, to their higher-level integration into lexical units and syntactic phrases, and ultimately to global comprehension. This hierarchical organization may be related to a hierarchy of temporal scales, from short-term phonetic analysis at a temporal scale of tens of milliseconds, to syllabic envelope modulations around 200 ms, and slower prosodic-syntactic phrases with durations of the order of magnitude of typically a second. Importantly, these temporal scales are found in all languages of the world, and, in particular, the regularity of syllabic rhythms has been the focus of a large number of studies (Ramus et al., 1999; Pellegrino et al., 2011; Ding et al., 2017).

This hierarchy of temporal scales finds a strong echo in the hierarchy of neuronal rhythms structuring the electrical activities of the brain, with characteristics that can be related to several cognitive functions (Buzsáki and Draguhn, 2004; Buzsaki, 2006; Fries, 2015). Although still a matter of debate, there is a growing consensus on the potential causal role of brain rhythms in the perception and understanding of speech (Poeppel and Assaneo, 2020). An influential model relating speech and brain rhythms has been proposed by Giraud and Poeppel (2012), in which the speech input would be initially parsed according to syllabic rhythm thanks to neural oscillatory processes in the theta band (4–8 Hz). Inside syllabic chunks, phonetic analysis would be conveyed by gamma oscillations at around 40 Hz. Further syntactic parsing would rely on lower frequency processes in the delta range (1–2 Hz). This model is embedded in the predictive coding framework (Rao and Ballard, 1999; Friston, 2005; Friston and Kiebel, 2009), which hypothesizes that the brain is inherently predictive, exploiting internal states to make inferences about upcoming sensory data. This framework provides a way to integrate top-down predictions with bottom-up information. Top-down information from various stages of the speech perception process would be fed back to lower processing stages, possibly exploiting the beta band (15–20 Hz) which is assumed to be a relevant channel for providing such descending predictions (Engel and Fries, 2010; Arnal, 2012; Arnal and Giraud, 2012; Sohoglu et al., 2012; Rimmele et al., 2018).

### 1.2. Neuro-Computational Models of Syllabic Segmentation

These theoretical proposals gave rise to a number of recent neuro-computational models of speech perception exploring the possibilities offered by neural oscillations to address issues related to speech segmentation. The common point between all these models is that they use a sensory, input-driven approach, where the slow modulations of the speech signal envelope would be tracked by endogenous cortical oscillations. This enables parsing speech into intermediate speech units such as syllables, which play an important role in the speech prosodic structure used to segment the continuous acoustic stream (Grosjean and Gee, 1987; Rosen, 1992; Kolinsky et al., 1995).

Following experimental evidence by Ghitza and Greenberg (2009) on the role of syllabic modulations as potential “packaging units” for further speech processing in the human brain, Ghitza (2011) presented the TEMPO model based on the “syllabic packaging” principle. In TEMPO, a lower auditory analysis stage is in charge of extracting syllabic parsing information, controlling the decoding process performed at a higher stage, in charge of phonetic decoding before lexical access. Syllabic parsing in TEMPO is based on a “theta oscillator” tracking modulations of the acoustic envelope in the 4–10 Hz frequency range typical of syllabic rhythm (Greenberg, 1999). Then, so-called theta-cycles are divided in a fixed number of sub-syllabic units (fixed at 4 in the first version of TEMPO, Ghitza, 2011), in charge of phonetic decoding (see also the model developed by Yildiz et al. (2013) for word recognition, using the same architecture though with 8 sub-syllabic units instead of 4). Theta-cycles would be generated in the auditory cortex (Ghitza, 2013), likely in superficial layers of auditory neurons (Giraud and Poeppel, 2012), potentially influenced by sensory-motor interactions with parieto-frontal regions in the dorsal cortical pathway (Morillon et al., 2010; Strauß and Schwartz, 2017).

While a computational version of TEMPO has not been tested on speech corpora to the best of our knowledge, neurocomputational models based on such principles have been implemented and tested in the past years. Firstly, Hyafil et al. (2015) introduced a spiking theta network model called Pyramidal Interneuron Theta (PINTH), by analogy with the so-called Pyramidal Interneuron Gamma (PING) model implementing a gamma network with bursts of inhibitory neurons following bursts of excitatory neurons (Jadi and Sejnowski, 2014). The PINTH model connects spiking excitatory and inhibitory neurons that synchronize at theta frequency (6–8 Hz), through slowly decaying inhibition, which leads to resonance behaviors in the corresponding frequency range. PINTH neurons are driven by the output of a (supposedly sub-cortical) auditory model receiving the speech acoustic stimulus as input. The model, applied to a corpus of real speech signals consisting of phonetically labeled English sentences (the TIMIT corpus; Garofolo, 1993), was shown to produce consistent theta bursts following syllabic onsets. Still, precise quantitative evaluation was not provided by the authors. Indeed, the syllabic alignment accuracy is said to remain “well above chance levels” (Hyafil et al., 2015, p. 5) but it seems to remain rather low when exposed to the variety of stimulation occurring in real speech.

Then, a new version proposed by Hovsepyan et al. (2020) replaces the spiking network in Hyafil et al. (2015) by a continuous model driven by coupled first-order nonlinear differential equations providing a resonant system—in the 3–8 Hz frequency range—driven by the envelope of the acoustic input. The model includes a systematic sequence of 8 phone decoders inside each syllable, as in Ghitza (2011) and Yildiz et al. (2013), together with a reset mechanism that automatically triggers an onset at the end of the last phone decoder (the 8th). This reset mechanism hence exploits predictions about the input spectrogram stored in memory, though no explicit fusion process exploiting temporal top-down timing predictions from higher linguistic levels. The model was tested on 220 sentences of the previously mentioned TIMIT database. It yielded only moderate syllabic detection accuracy, estimated at 53 %, by measuring the percentage of time when a given syllable labeled as such in the database corresponded to the theta-cycle detected by the model.

Hence, altogether, the performance of these models seems rather limited. At least three sets of reasons could explain such low performance level. Firstly, these models are based on oscillators which drive the models into a predictive mode in which timing regularity is the rule and irregularity the exception, while natural speech timing is known to be far from isochronous. This is likely to limit performance, as displayed by the large trend for too regular theta-syllable durations compared with real syllabic durations in both Hyafil et al. (2015, Figure 1B) and Hovsepyan et al. (2020, Supplementary Figure 2). Secondly, event detection strongly depends on the amplitude and saliency of envelope modulations, which is modulated by phonetic context (e.g., smaller modulations for nasal stops, liquids or semi-vowels than for unvoiced stops in vowel-consonant-vowel sequences) and by the strength of articulation (e.g., smaller modulations for hypo-articulated speech compared with hyper-articulated speech, see Lindblom, 1990). Finally, we could expect event detection to be largely degraded in adverse conditions, such as for instance, superimposed acoustic noise likely to produce a number of spurious envelope fluctuations resulting in mistakenly detected events. Many studies have shown that, in these conditions, performance is negatively impacted, for listeners of all ages (Wong et al., 2008; Anderson et al., 2010, 2011).

In this context, purely bottom-up algorithms for event detection and syllabic parsing do not appear sufficient for efficient speech processing. Instead, it seems likely that top-down predictions exploiting the listener's knowledge of the timing of natural speech (e.g., lexical or prosodic information) could improve the efficiency of purely bottom-up segmentation (Davis and Johnsrude, 2007). Indeed, clear evidence for the role of top-down timing predictions has been recently provided by Aubanel and Schwartz (2020). Their study showed that speech sequences embedded in a large level of noise were better processed and understood by listeners when they were presented in their natural, irregular timing than in a timing made isochronous without changing their spectro-temporal content. The strong benefit in intelligibility displayed by natural syllabic timing, both in English and in French, was interpreted by the authors as evidence for the role of top-down temporal predictions for syllabic parsing.

### 1.3. Toward a New Neurally-Inspired Computational Model Introducing Top-Down Temporal Predictions for Syllabic Segmentation

The objective of the present neurally-inspired computational model is to address, for the first time to our knowledge, the question of the fusion of bottom-up and top-down processes for speech syllabic segmentation. We address this question in a Bayesian computational framework, which enables to efficiently introduce, conceptualize and compare computational processes expressed in a unified probabilistic formalism (Bessière et al., 2008). The combination of bottom-up information extraction and top-down predictions from higher linguistic levels is actually not new. Indeed, it is at the heart of all modern speech recognition architectures, be they classical Hidden Markov Models (HMM) in which bottom-up acoustic cues are associated to top-down state transition probabilities in phonetic decoding or word recognition (Rabiner et al., 1989; Gales and Young, 2008) or more sophisticated architectures such as hierarchical HMMs (Murphy, 2002) or multi-scale HMMs enabling to incorporate hierarchical linguistic structures in language processing (Eyigöz et al., 2013); and of course recent neural speech recognition models including recurrent architectures implementing top-down feedback in the decoding process (see Graves et al., 2013; or a recent review in Kim et al., 2020). In the field of psycholinguistics also, since the pioneer development of TRACE (McClelland and Elman, 1986), the question of the role of feedback processes in speech perception and comprehension has been the focus of intense discussions (Norris, 1994), and led to many developments in Bayesian framework (Norris and McQueen, 2008). Recent findings confirm that recurrence plays a crucial role in perceptual processing in the human brain (e.g., Kietzmann et al., 2019; Spoerer et al., 2020). Still, while the importance of top-down predictions has been largely discussed in the literature, it has been mainly focused on the mechanisms involved in the decoding process, though not on the segmentation process *per se*. This is precisely the objective of the present work.

For this aim, we introduce COSMO-Onset, a variant of the COSMO framework developed over the years to simulate speech communication processes in a perceptuo-motor framework (Moulin-Frier et al., 2012, 2015; Patri et al., 2015; Laurent et al., 2017; Barnaud et al., 2018). The present variant does not incorporate at this stage the whole perceptuo-motor loop developed and studied in previous COSMO papers. Instead, it concentrates on the auditory pathway, detailing two mechanisms of interest for the present study: first, a hierarchical decoding process combining the phonetic, syllabic and lexical levels, and, second and most importantly in the present context, a syllabic parsing mechanism based on event detection, operating on the speech envelope. COSMO-Onset is a Bayesian speech perception model associating a decoding module to decipher the spectro-temporal content of the speech input and a temporal control module enabling to control how the speech input is segmented into constituent linguistic units. The decoding module has a hierarchical structure similar to classical psycholinguistic models like TRACE (McClelland and Elman, 1986), with three layers of representations (acoustic features, syllable and word identity) usually considered in the context of isolated word recognition. The temporal control module associates a bottom-up mechanism for syllabic onset detection with an original top-down mechanism for syllabic onset prediction, involving lexical temporal knowledge. The bottom-up onset detection process does not rely on oscillatory models as in Ghitza (2011), Hyafil et al. (2015), and Hovsepyan et al. (2020), but features instead a simple algorithm based on detecting loudness increases in the envelope, and regulating it by a refractory period. Indeed, the focus of present model is set on studying the way bottom-up and top-down information could be efficiently combined for syllabic parsing.

With this model, we explore the dynamics of speech segmentation resulting from the combination of such bottom-up and top-down temporal mechanisms. It is crucial at this stage to make clear the novelty of the present study, together with some explicit limitations of its perspective. Indeed, the Bayesian formalism is seldom used in the context of such an event detection task, and much care has been taken in this study to conceptualize the way temporal fusion could occur and be expressed in Bayesian terms. Therefore, the contribution of the COSMO-Onset model is, at this stage, rather conceptual and focused more on principles than on realistic simulations. Nevertheless, the fusion architecture has been developed in relation with the observed weaknesses of the existing computational models presented in the previous section. Hence, the paper does involve a set of principled simulations addressing two potential problems for a purely bottom-up event detection process.

Firstly, we consider the case of attenuated envelope modulations associated to, for instance, specific phonetic contexts or hypo-articulated speech. This leads to a situation in which, compared to a “nominal” configuration where envelope modulations enable a bottom-up mechanism to perform efficiently, some events would be missed by the bottom-up processing branch. To deal with such perturbation, we consider a so-called OR fusion process, in which detected onsets could be provided either by the bottom-up processing of acoustic envelope or by temporal predictions from a top-down process capitalizing on the temporal structure of stored items in the lexicon and their ongoing recognition. Secondly, we consider the case of noisy conditions where spurious envelope modulations would appear and lead to spurious events in the bottom-up processing branch. To deal with such degradation, we consider another fusion process, that is an *AND* fusion, in which events would be detected only if bottom-up detection and top-down prediction co-occur more or less in a synchronous manner.

Therefore, we formulate the following predictions. First, we expect a model based solely on bottom-up information to perform well in nominal conditions. Second, in the case of adverse conditions, we expect top-down predictions of onset events to yield robust performance, that is, to maintain performance despite perturbations. In conditions such as hypo-articulated speech, we expect a model based on the *OR* fusion model to be robust; in conditions such as with a noisy environment, we expect a model based on the *AND* fusion model to be robust.

In the next section, we present the model architecture, and we describe its two components, the spectro-temporal decoding module and the temporal control module exploiting a fusion process between sensory-driven bottom-up segmentation and knowledge-driven top-down onset prediction. In Section 3, we present our simulations, which rely on a representative set of “toy” stimuli and situations, elaborated for the purpose of this preliminary study to assess the various components of the model. More precisely, we highlight the role of the top-down onset detection component, systematically comparing performance of the pure bottom-up process with the *AND* and OR fusion processes incorporating top-down temporal predictions. Section 4 presents the corresponding simulation results. Finally, the general discussion in Section 5 addresses potential neurocognitive predictions that could emerge from the simulations with the *AND* and OR fusion models, and provides perspectives for more elaborate simulations toward the processing of realistic speech inputs.

## 2. Model Architecture

COSMO-Onset is a hierarchical probabilistic model. It is mathematically specified by a joint probability distribution, defined by its decomposition into a product of probability distributions, some of which are simplified, thanks to conditional independence hypotheses. The complete mathematical definition of the model is provided in Supplementary Materials (see sections A.1–A.4); here, instead, we describe the overall structure of the model, and its resulting simulation of spoken word recognition processes.

**Figure 1:**
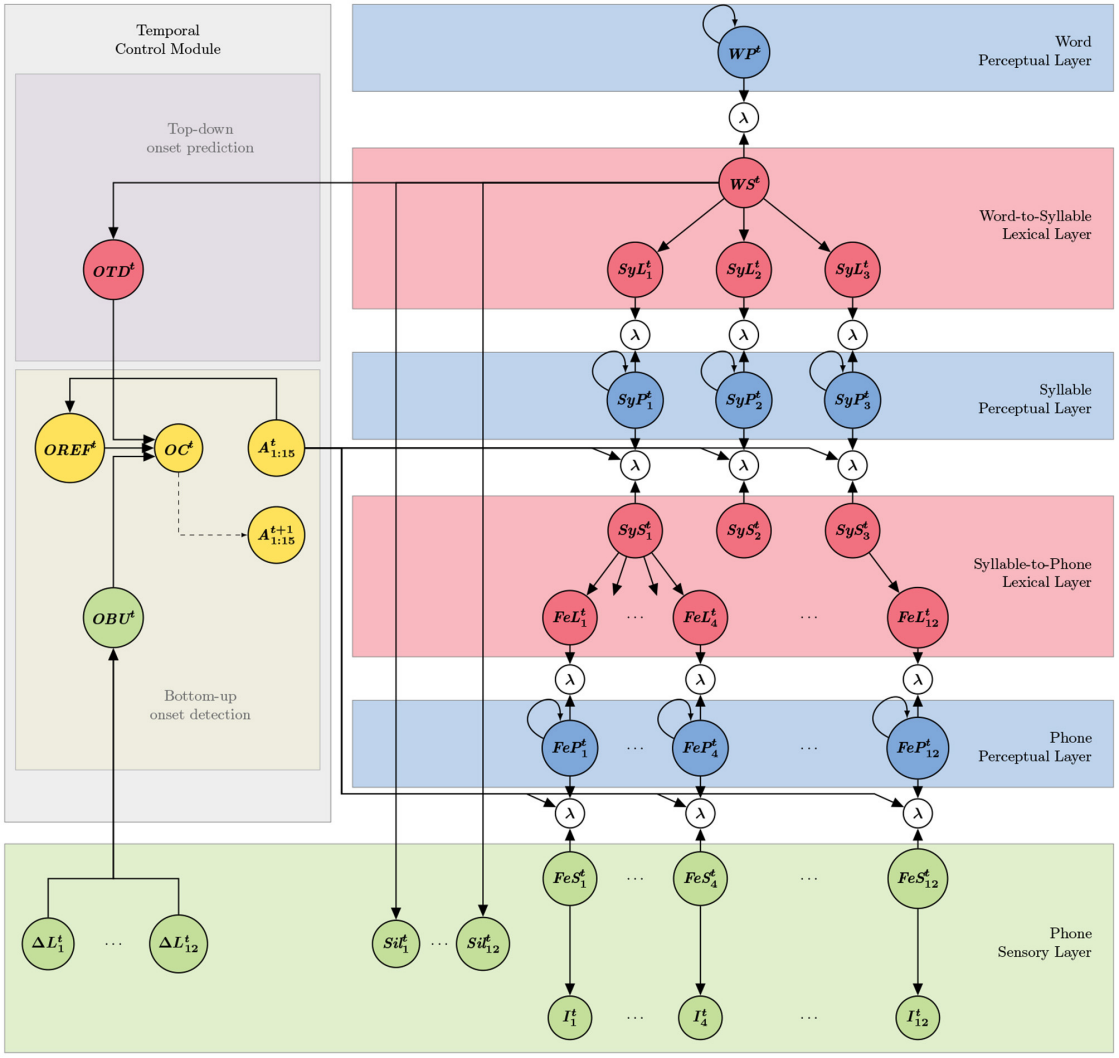
Graphical representation of the COSMO-Onset model. Variables of the model are represented as nodes (a summary of variable names and their interpretation is available in Table 1). Subscripts indicate position in sequential parsing of the input into linguistic unit, and superscripts indicate time instant. For instance, ${\text{SyP}}_{1}^{t}$ is the variable related to the first syllabic decoder at time *t*. Probabilistic dependencies between variables are represented by arrows: there is an arrow from node *X* to node *Y* if *X* is a “parent node” of *Y*, that is to say, *X* appears as a conditioning variable of *Y* in a term [e.g., the arrow from ${\text{SyS}}_{1}^{t}$ to ${\text{FeL}}_{1}^{t}$ represents the term $P\left({\text{FeL}}_{1}^{t}|{\text{SyS}}_{1}^{t}\right)$]. Self-looping arrows denote dynamical models, that is to say, a variable that depends on the same at the previous time step [e.g., there is a term *P*(*WP*^*t*^ ∣ *WP*^*t*−1^)]. The dotted arrow between node *OC*^*t*^ and node $A_{1:15}^{t+1}$ is not a probabilistic dependency, and represents instead a decision process (i.e., the probability that *OC*^*t*^ is *True* is compared to a threshold, and this conditions variables $A_{1:15}^{t+1}$). Sub-models are represented as colored rectangular blocks, to assist model description (see text for details). Portions of the model, specifically, some “phone branches” are not shown, for clarity.

**Figure 2:**
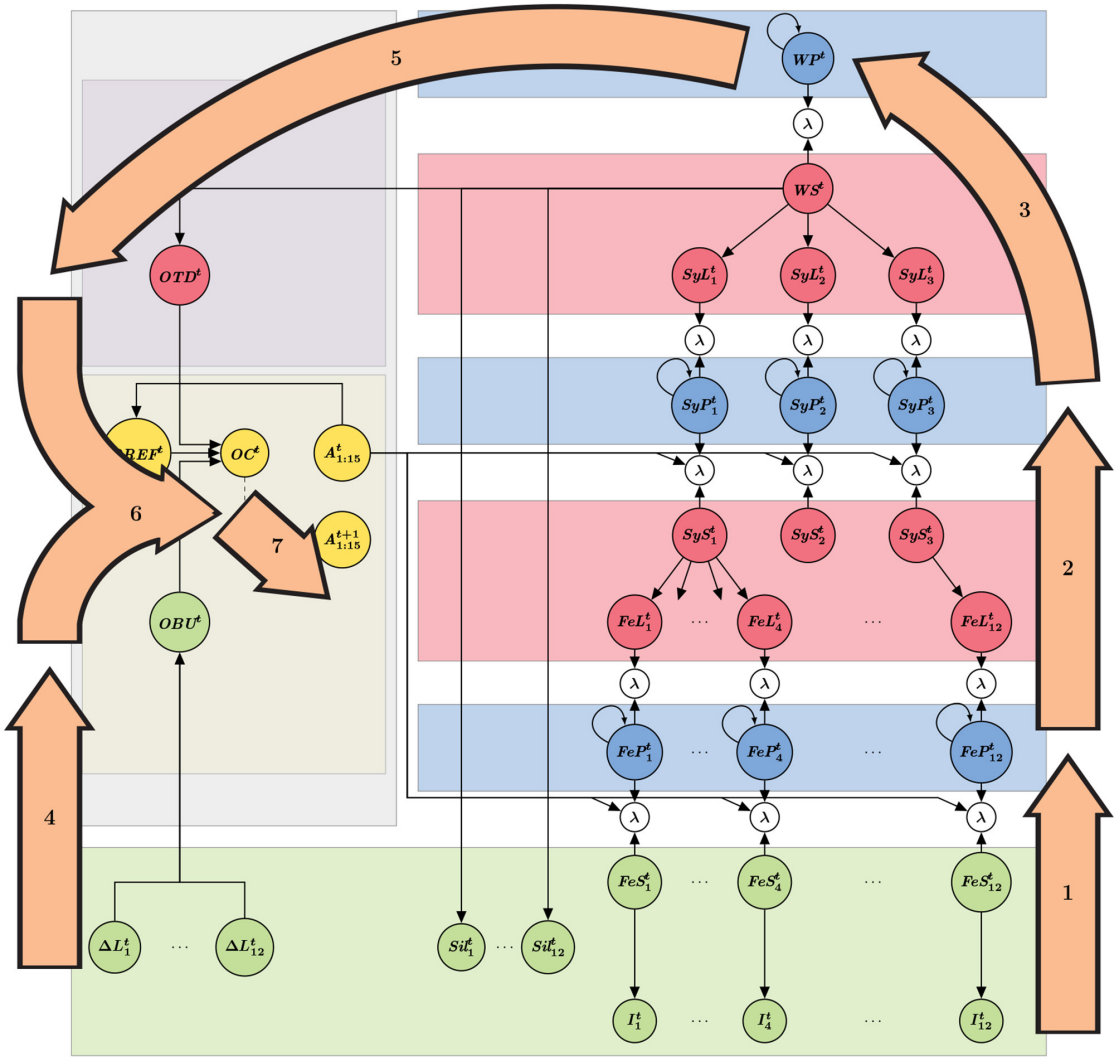
Graphical representation of computation steps to perform word decoding and onset detection. Orange arrows indicate inference steps, labeled in the order they are performed, and roughly superposed to the relevant portions of the model for each step (see Figure 1). Step 1 is feature decoding, step 2 is syllable decoding, step 3 is word decoding; these steps are performed in a purely feedforward manner in the decoding module (i.e., no lexical feedback to bias syllable or feature decoding). In the blue nodes, incoming input information is combined with information of the previous time-step. Step 4 is bottom-up onset detection from the input, step 5 is lexical, top-down onset prediction, step 6 is the combination of the results of steps 4 and 5 (applying either the *AND* or the *OR* operator). Finally, step 7 is a decision process on the combined onset probability; when an onset is detected, the states of decoding gates (open or closed) are changed. Notice that it is precisely at step 7 that the implementation shifts toward the next time step, thus breaking the probabilistic dependency cycle to avoid complex computational loops.

The probabilistic dependency structure, which results from the conditional independence assumptions used to define the model, can be interpreted as the architecture of the model, and can be graphically represented (see Figure 1). The left part of the schematic representation of the model (gray rectangle in Figure 1) is the “temporal control” submodel, whereas the rest (right part of the schema) constitutes the “decoding” portion of the model. Applying Bayesian inference to the model architecture provides computing steps for simulating word recognition and onset detection; these steps are illustrated on the model structure in Figure 2. Finally, Table 1 provides the summary of variable names and their interpretation. We now describe first a panorama of the general principles and functioning of the model, and second, each of the two submodels.

**Table 1:** Summary of symbols: variable names and their interpretation.

**Variables for acoustic signal description**	
$I_{1:12}^{1:T}$	Spectral contents of the acoustic signal Input (F1, F2 formants)
$\Delta L_{1:12}^{1:T}$	Derivative of the Loudness of the acoustic signal
$Sil_{1:12}^{1:T}$	SILent portions of the acoustic signal (Boolean)
**Variables for linguistic content**	
$FeS_{1:12}^{0:T}$, $FeP_{1:12}^{0:T}$, $FeL_{1:12}^{0:T}$	Phones (i.e., FEatures), respectively from Sensory decoding, in phone Perceptual accumulators and from Lexical prediction
$SyS_{1:3}^{0:T}$, $SyP_{1:3}^{0:T}$, $SyL_{1:3}^{0:T}$	SYllables, respectively from Sensory decoding, in syllabic Perceptual accumulators and from Lexical prediction
**WS**^0:*T*^, **WP**^0:*T*^	Words, respectively from Sensory decoding and in the word Perceptual accumulator
**Variables for controlling information flow in the model**	
$\lambda FeSP_{1:12}^{1:T},\ldots$, $\lambda FePL_{1:12}^{1:T},\ldots$	Coherence or controlled coherence variables, connecting layers of the model
$A_{1:15}^{1:T}$	Control variables modulating information flow (opening, closing and sequencing phone and syllable perceptual accumulators)
**Variables for onset detection**	
**OTD**^1:*T*^, **OBU**^1:*T*^, **OREF**^1:*T*^, **OC**^1:*T*^	Onset detectors (Boolean), respectively from TD knowledge, BU sensory decoding, REFractory period inhibition and Combined result

### 2.1. General Principles

As shown in Figure 1, the overall structure of the decoding portion of the model consists in six layers, connected by Boolean variables called “coherence variables” (represented by λ nodes in Figure 1). These can be seen as “probabilistic glue,” allowing merging, in a mathematically principled manner, probability distributions over the same domains (Gilet et al., 2011; Bessière et al., 2013). During inference, these coherence variables are used to choose how probabilistic information propagates into the model; in that sense, they can be interpreted as “Bayesian switches.” First, they can be “closed,” so that between two connected variables information propagates through them. Mathematically, this corresponds to assuming that the value of the coherence variable is known and equal to 1, and it yields a product of the probability distributions of the variables connected by the coherence variable (whatever these probability distributions). Second, a Bayesian switch can be “open,” by ignoring its value during inference; this results in disconnecting the corresponding portions of the model connected by the coherence variable, through a marginalization process that can be shown to simplify. Technical details can be found elsewhere (Gilet et al., 2011, see also section A.5 in the Supplementary Material).

Some of the coherence variables in the decoding module (the ones with input arrows coming from node $A_{1:15}^{t}$ in the temporal control module (see Figure 1) are further “controlled” (Phénix, 2018), that is to say, they allow controlling in a gradual manner the propagation of probabilistic information, from the phone to the syllable to the word perceptual layers. Where coherence variable can be interpreted as “Bayesian switches,” controlling information flow in an all-or-none manner, controlled coherence variables can be interpreted as “Bayesian potentiometers,” thanks to their gradual control of information propagation. Technically, this is done by connecting a probability distribution over the control variable, which is Boolean, to the coherence variable. The probability that the control variable is “True” then modulates the amount of probabilistic information propagation (see section A.5 in the Supplementary Material).

In the context of the COSMO-Onset model, we therefore use controlled coherence variables to modulate, over time, information flow in the model (see variable $A_{1:15}^{t}$ in Figure 1). This allows to modify dynamically, during perception, which portion of the model receives and processes sensory evidence. In other words, variables $A_{1:15}^{t}$, which are the main output of the temporal control module, are used to explicitly “open” or “close” channels through which probabilistic information propagates in the model.

More precisely, we employ such a mechanism to control information flow between one “word decoder” (variable *WP*^*t*^), 3 “syllabic decoders” (variables ${\text{SyP}}_{1}^{t}$, ${\text{SyP}}_{2}^{t}$ and ${\text{SyP}}_{3}^{t}$) and 12 “phone decoders” (variables ${\text{FeP}}_{1}^{t}$ to ${\text{FeP}}_{12}^{t}$), so that the word decoder includes a sequence of 3 syllabic decoders and each syllabic decoder includes 4 phone decoders. In other words, control variables $A_{1:15}^{t}$ control the temporal windows during which phone and syllable perceptual variables receive sensory evidence to process. This allows implementing the sequential activation of phone and syllabic decoders, that is to say, phone and syllabic parsing.

The purpose of the temporal control module is thus exactly to control syllabic and phone parsing. To do so, it computes, at each time step, the probability that there would be a syllabic onset event, that is, the probability that a new syllable begins in the acoustic input. When this probability passes a threshold, the system decides there was an onset, which has two main effects (see Figure 1, dotted arrow). First, the currently “activated” phone and syllable decoders stop receiving sensory input from the stimulus or lower-level layers. Second, the next syllabic decoder, in a sequential order, is activated, along with its first phone decoder. In contrast, phone decoders process input for a fixed time and then activate the next one. Therefore, our model segments the continuous speech stream into linguistic units of varying lengths at the syllabic level. Consequently, the model can handle words that have a varying number of syllables, syllables that have a varying number of phones, and phones that have a varying number of iterations (since the last phone decoder of a syllable can be “deactivated” before completion, as does the last syllable decoder of a word). We note, as mentioned in section 1.2, that previously proposed models also feature such mechanisms, of sequential activation and deactivation of syllabic (Ghitza, 2011; Hyafil et al., 2015; Hovsepyan et al., 2020) and phone decoders (Yildiz et al., 2013).

### 2.2. Decoding Module

The decoding module of the COSMO-Onset model is inspired both by the BRAID model (Bayesian model of Word Recognition with Attention, Interference and Dynamics) of visual word recognition (Phénix, 2018; Ginestet et al., 2019) and by the classical, three-layer architecture of models of spoken word recognition, such as the TRACE model (McClelland and Elman, 1986). It can also be construed as a hierarchical (multi-layered) dynamic Bayesian network (Murphy, 2002), with an external component to control information propagation.

Three representation layers are featured in the model, with internal representation of phones, of syllables, and of words. Each is associated with a series of probabilistic dynamic models (i.e., Markov-chain-like probabilistic terms, colloquially referred to as “decoders” above), which allow continuous accumulation of sensory evidence about the representation domain they consider. Information gradually decays from these Markov chains, to ensure return to their respective initial states in the absence of stimulation. However, information decay rate is set to a low value, to basically ensure that the result of sensory evidence accumulation is maintained and remains available for the whole duration of processing a given word. Therefore, these Markov chains essentially provide perceptual models about phonetic states (“phones” in the following), syllables and words (see blue rectangles in Figure 1), central to phone, syllable and word recognition.

Two lexical layers (red rectangles in Figure 1) feature probabilistic terms to define “transformation terms,” that is to say, knowledge about how one representational space maps onto another. The word-to-syllable lexical layer describes how known words are composed of known syllables [with terms of the form $P\left({\text{SyL}}_{i}^{t}|{\text{WS}}^{t}\right)$], whereas the syllable-to-phone lexical layer describes how known syllables are composed of known phones [with terms of the form $P\left({\text{FeL}}_{j}^{t}|{\text{SyS}}_{i}^{t}\right)$]. The final layer of the decoding portion of the model (green rectangle in Figure 1) maps phones onto sensory input, and more precisely, represents knowledge about how known phones correspond to acoustic signals [with terms of the form $P\left(I_{j}^{t}|{\text{FeS}}_{j}^{t}\right)$]. It also contains the complete description of the acoustic input, with both its spectral and amplitude (loudness) contents.

With these six layers connected by coherence variables, so that sensory information can enter the model and propagate, we can then simulate word recognition with the decoding portion of the model, that is to say, compute the probability distribution over variable *WP*^*t*^, at each iteration *t*, given the acoustic stimulation, as described by variables $I_{j}^{t}$, ${\text{Sil}}_{j}^{t}$ and $\Delta L_{j}^{t}$. Because of the complex structure of the model, with its hierarchically layered Markov-chains, Bayesian inference results in complex computations, involving both feed-forward (from acoustic input to word space) and feed-back (from word space to acoustic input) propagation of information. However, in the current study, we approximate these, considering word recognition in the decoding module as a purely feed-forward process (in contrast with our main focus of this study, that is, the inference in the temporal control module, which features both bottom-up and top-down components; see below). Word recognition, in the decoding module, is thus based on phone recognition (Figure 2, step 1) activating syllable recognition (Figure 2, step 2), and finally word recognition *per se* (Figure 2, step 3). These steps are based on time-varying computations of probability distributions, respectively over the phone perceptual variables, the syllable perceptual variables and the word perceptual variables.

### 2.3. Temporal Control Module

The “bottom-up” portion of the temporal control module assesses the probability of syllabic onset events by relying on the temporal cues that can be extracted from the speech envelope (step 4, in Figure 2). This has been largely discussed in the literature and several models of syllable onset and boundary detection have been proposed (Mermelstein, 1975; Ghitza, 2011; Hyafil et al., 2015; Räsänen et al., 2018; Hovsepyan et al., 2020). These models process the speech envelope, either in search of rapid increases or decreases (troughs) in the energy of the speech envelope. In the COSMO-Onset model, syllable onset detection from the stimulus is based on tracking the rapid increase of energy in the speech envelope. If such an increase is detected for several successive time steps, and if the corresponding increase exceeds a given threshold, then the probability of an onset events gets high (terms of the form $P\left({\text{OBU}}^{t}|\Delta L_{j}^{t}\right)$, see Figure 1).

The “top-down” portion of the temporal control module relies on lexical knowledge about word composition. This lexical knowledge associates each word of the lexicon to a sequence of syllables, each of a known composition, thus of known duration. Therefore, the model incorporates knowledge about the “canonical” instants at which syllabic onset can be expected, for each word [term *P*(*OTD*^*t*^ ∣ *WS*^*t*^)]. During word recognition, this lexical prediction of onset events is combined with the ongoing computation of the probability distribution over words, so that words contribute to syllabic onset prediction according to their current probability (step 5, in Figure 2).

The next component of the temporal control module is a fusion model between the bottom-up detection and top-down prediction of onset events (term *P*(*OC*^*t*^ ∣ *OTD*^*t*^*OBU*^*t*^*OREF*^*t*^), step 6, in Figure 2). We define two ways of combining the two pieces of information, through two fusion “operators,” the *AND* and the *OR* operators. They are both mathematically defined as particular products of the probability distributions provided by the top-down and bottom-up components. Nevertheless, they can easily be interpreted: with the *AND* operator, the temporal control module decides that there is an onset event if both the bottom-up and top-down components agree that there is one; in contrast, with the *OR* operator, the temporal control module decides that there is an onset event if at least one component suggests that there is one.

The final component of the “bottom-up” portion of the temporal control module implements a refractory period (variable *OREF*^*t*^, also within step 6, in Figure 2). If an onset event was detected, this refractory mechanism prevents successive detection for a given time-window. This is inspired by well-known properties of the dynamics of oscillators in speech processing, that prevent the firing of successive onsets in the same oscillation period, classically observed in the theta band (Schroeder and Lakatos, 2009; Wyart et al., 2012). This is also a classical feature of previous models (Hyafil et al., 2015).

The computed probability that there is an event [*P*([*OC*^*t*^ = True]∣*OTD*^*t*^*OBU*^*t*^*OREF*^*t*^)] is then compared with a decision threshold: if it exceeds this threshold, an onset event is considered detected, which closes and opens the appropriate phone and syllable decoders of the decoding module, for the next simulation time step. This is represented, in Figure 1, by the dotted arrow between nodes *OC*^*t*^ and ${\text{A}}_{1:15}^{t+1}$ (step 7, in Figure 2).

## 3. Material

### 3.1. General Principles

The present contribution is focused on the definition and assessment of the major principles of the COSMO-Onset model. For this aim, and since we are still far from a complete implementation and evaluation on real speech stimuli, we have defined a set of highly simplified material, easily tractable but still enabling to test all the different components of the model. This “toy” material hence respects a compromise between two antagonist requirements: being sufficiently varied to display a variety of configurations for the model and being sufficiently simple to enable simple simulations easy to interpret at this initial stage of development.

### 3.2. Linguistic Material

The linguistic material we consider in this first study is made of isolated words with a variable number of syllables from 1 to 3, and syllables made of either a single vowel (a V syllable) or a sequence of a consonant and a vowel (a CV syllable). We consider a set of 3 vowels /a i u/ and 2 plosive consonants /p t/. Furthermore, we defined a lexicon of 28 toy words, at most tri-syllabic, the list of which is provided in column 2 in Table 2.

**Table 2:** List of the 28 words of the lexicon together with their “phonetic” content.

**Word type**	**Word**	**Phone sequence**	**Duration**
Monosyllabic	“*a”*	*a*-#	150
	“*pa”*	*p*-@-*a*-#	200
	“*pi”*	*p*-@-*i*-#	200
	“*pu”*	*p*-@-*u*-#	200
	“*ta”*	*t*-@-*a*-#	200
	“*ti”*	*t*-@-*i*-#	200
	“*tu”*	*t*-@-*u*-#	200
Bi-syllabic	“*apa”*	*a*-*p*-@-*a*-#	300
	“*ata”*	*a*-*t*-@-*a*-#	300
	“*ipi”*	*i*-*p*-@-*i*-#	300
	“*iti”*	*i*-*t*-@-*i*-#	300
	“*upu”*	*u*-*p*-@-*u*-#	300
	“*utu”*	*u*-*t*-@-*u*-#	300
	“*papa”*	*p*-@-*a*-*p*-@-*a*-#	350
	“*pata”*	*p*-@-*a*-*t*-@-*a*-#	350
	“*patu”*	*p*-@-*a*-*t*-@-*u*-#	350
	“*pipi”*	*p*-@-*i*-*p*-@-*i*-#	350
	“*pita”*	*p*-@-*i*-*t*-@-*a*-#	350
	“*tata”*	*t*-@-*a*-*t*-@-*a*-#	350
	“*tatu”*	*t*-@-*a*-*t*-@-*u*-#	350
	“*tuti”*	*t*-@-*u*-*t*-@-*i*-#	350
Tri-syllabic	“*apata”*	*a*-*p*-@-*a*-*t*-@-*a*-#	450
	“*apiti”*	*a*-*p*-@-*i*-*t*-@-*i*-#	450
	“*iputu”*	*i*-*p*-@-*u*-*t*-@-*u*-#	450
	“*utatu”*	*u*-*t*-@-*a*-*t*-@-*u*-#	450
	“*patata”*	*p*-@-*a*-*t*-@-*a*-*t*-@-*a*-#	500
	“*patati”*	*p*-@-*a*-*t*-@-*a*-*t*-@-*i*-#	500
	“*tapatu”*	*t*-@-*a*-*p*-@-*a*-*t*-@-*u*-#	500

*Column 1 provides the grouping of words according to their number of syllables. Column 2 provides the name of each word, corresponding to its phonological content. Column 3 provides the phonetic content of each word, that is, the sequence of acoustic phones. Column 4 provides the corresponding duration of the model input, in simulated time steps*.

### 3.3. Phonetic Material

At the acoustic and phonetic level, we represent syllables by sequences of phones with a maximum number of 4 phones per syllable (in the same vein as in Ghitza, 2011). The sequence of phones for the 28 words in the lexicon is provided in column 3 in Table 2. For example, the word “*pata”* is composed of a sequence of 7 phones *p*-@-*a*-*t*-@-*a*-#, the content of which will be described in the following of this section. Altogether, the constraints on the maximal number of syllables per word (3) and phones per syllable (4) match with the decoding structure of the current COSMO-Onset implementation (see Figure 1), respectively in the word-to-syllable lexical layer and syllable-to-phone lexical layer.

**Figure 3:**
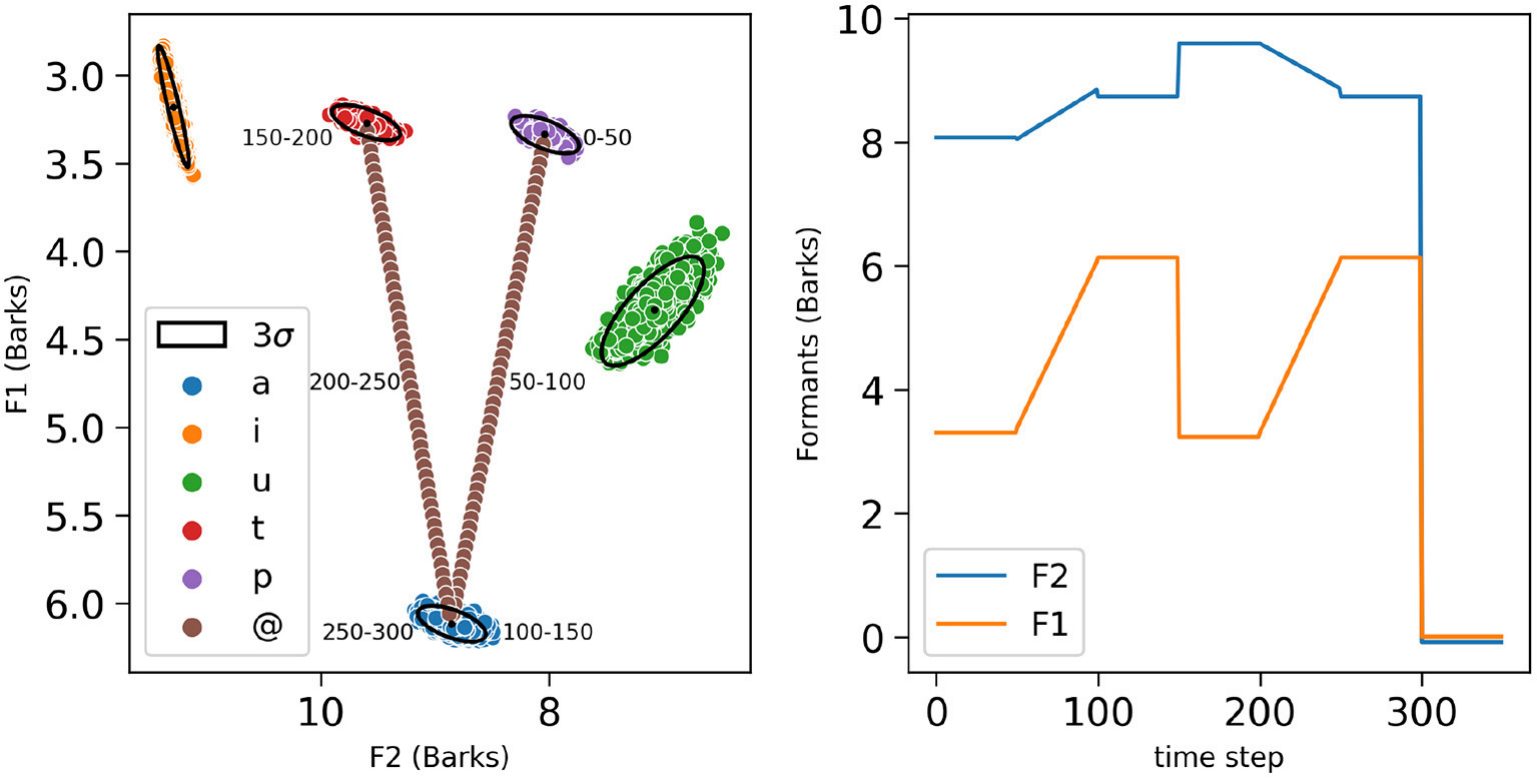
**(Left)** Phones of the lexicon represented on a two-dimensional space with the second formant F2 on the *x*-axis from right to left and the first formant F1 on the *y*-axis from top to bottom, as is classical in phonetic displays. Phones associated with phonemes /a/, /i/, /u/, /t/, and /p/ are, respectively, represented by blue, yellow, green, red and purple colored dots. The trajectory of the simulation of the word “pata,” is also displayed. The annotations correspond to the different corresponding time steps for each constituent phone of the word “pata,” with one sample of the phone *p* from 0 to 50 time steps, the transitional phone @ between the phones *p* and *a*, from 50 to 100 time steps, along a linear transition joining the barycenters of the two phone categories (brown dots), one sample of the phone *a* from 100 to 150 time steps, one sample of the phone *t* from 150 to 200 time steps, the transitional phone on 50 time steps, and one sample of the phone *a* from 250 to 300 time steps. For each phone, are also shown the mean (black dot) and the 3 standard-deviation ellipse of the bi-variate normal distribution best fitting the data points (black ellipses). **(Right)** Example of formant inputs (*y*-axis, in orange for F1, in blue for F2) used for the word “*pata*,” as a function of simulated time steps (*x*-axis).

Vowel and plosive phones in our simulations are acoustically represented as sets of pairs of formants *(F1, F2)* in Barks, a subjective perceptual scale (Zwicker, 1961; see Figure 3). While it is classical to characterize vowels by their first two formants (Fant, 1970), it is less classical to use formant values for plosives (although, see Schwartz et al. (2012) for a characterization of plosives by formant values). More precisely, the formant values for the considered vowels are gathered from a dataset obtained using VLAM, the Variable Linear Articulatory Model (Maeda, 1990; Boë and Maeda, 1998). It contains a large set of synthetic acoustic samples for all oral French vowels, and we only used the data points for the vowels /a i u/, respectively corresponding to phones *a, i* and *u* in the following, which amount to 15,590 samples. To this vowel set, we added 1,000 points for the phones *p* and *t* associated to consonants /p t/, 500 each, supposed to lie in the *(F1, F2)* space between *i* and *u, p* close to the back rounded *u* and *t* close to the front *i* (Schwartz et al., 2012).

For the syllables formed by two different phonemes (in the present simulations, C followed by V), in order to simulate formant transitions (Lindblom and Studdert-Kennedy, 1967; Stevens and Klatt, 1974; Dorman et al., 1975), we defined linear transitions between the phones associated to the constituent phonemes. An example of a transition between phones *a* and *u* is depicted in Figure 3. Transitions are denoted by the phone symbol @, both in descriptions of the stimuli used in the simulations, but also as a value in the phone space in the model.

Finally, in the present simulations, each word input consists in a phone sequence ending with an “end of sequence” marker, to signal silence in the acoustic signal. Silence is denoted by the phone symbol #, here again, both in descriptions of the stimuli and as a possible phone to be recognized by the model. An example of formant sequence used as input for the bi-syllabic word “*pata*” is shown in Figure 3 right.

All these formant data distributions for each phone are used to obtain the parameters of the sensory models, that is, the probability distributions over acoustic input for each phone category [term $P\left(I_{j}^{t}|\left[{\text{FeS}}_{j}^{t}=f\right]\right)$]), and more precisely, their parameters, i.e., the means and co-variances of the Gaussian distributions for each phone in the lexicon (see Figure 3, black dots and black ellipses). In the case of the end-of-sequence marker #, it is arbitrarily mapped with formants normally distributed around the origin of the 2D formant space; such an arbitrary value is well outside of the meaningful formant descriptions for the vowels and the consonants of the lexicon, and thus silence is “easily recognized.”

### 3.4. Phone Duration and Loudness Profiles

In the current simulations, we consider that all phones have a constant duration of 50 ms, that is, 50 “time steps” (we keep the description of simulations in terms of time steps in the following, acknowledging that they would correspond to ms for application to real acoustic inputs). Nevertheless, syllables have variable duration since they have a variable number of phones. This number varies from 1 to 4: 1 for a non-terminal syllable made of a single vowel (e.g., the initial syllable in word “*apata”*), 2 if the vowel is followed by a final silence # (e.g., in the monosyllabic word “*a”*), 3 for a CV syllable with a phone for C and a phone for V connected by a transitional phone @, and 4 in a CV syllable that ends a word, because of the end-of-sequence phone. Accordingly, the duration of each word stimulus is displayed in column 4 in Table 2. For example, the word “*pata”* is composed of 50 ms of the phone *p*, followed by 50 ms of the transitional phone @, followed by 50 ms of the phone *a*, and so on, to end with 50 ms of the “end of word” marker #.

In addition to its description in terms of temporal sequence of phones, each syllable is characterized by a loudness profile *L* which provides the input to the temporal control module for syllable segmentation. Loudness represents the auditory evaluation of acoustic intensity at a given time, resulting from sensory processing of the acoustic signal envelope. This can be seen as capturing the variations of energy of the acoustic signal. In our simulations, loudness values are normalized between 0 and 1. Positive values of the local derivatives of loudness are used to define onset events.

**Figure 4:**
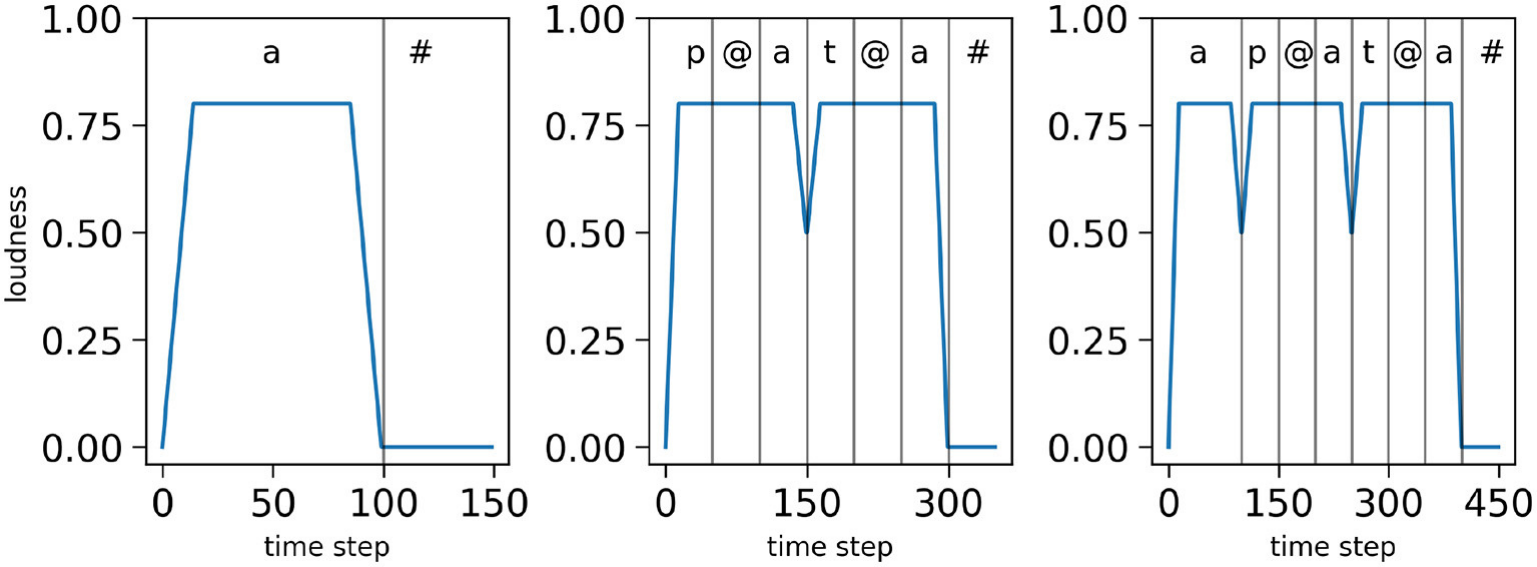
Examples of loudness variations for three input sequences: the word “*a*” **(left)**, the word “*pata*” **(middle)** and the word “*apata*” **(right)**. Simulated time is on the *x*-axes, normalized loudness (arbitrary units) is on the *y*-axes. The vertical bars and top annotations refer to the associated phonetic content of the stimulus.

The loudness profiles used in this study are simplified, and serve to illustrate and capture the fact that there are syllabic energy fluctuations in real speech with, generally, rapid increase at syllable onsets and gradual decrease toward syllable offsets (in-between, almost anything can happen). In Figure 4, we display examples of loudness profiles we use in the simulations, respectively for the mono-syllabic word “*a*,” composed of one vowel (Figure 4 left), for the bi-syllabic word “*pata*,” composed of 2 CV syllables (Figure 4 middle) and for the tri-syllabic word “*apata*” composed of 3 syllables (one V and 2 CV syllables, Figure 4 right).

### 3.5. Paradigms for Test Conditions

We explored various test conditions for the model, in order to assess and illustrate the interaction between the bottom-up onset detection and the top-down onset prediction mechanisms, with the stimuli configured as presented above.

**Figure 5:**
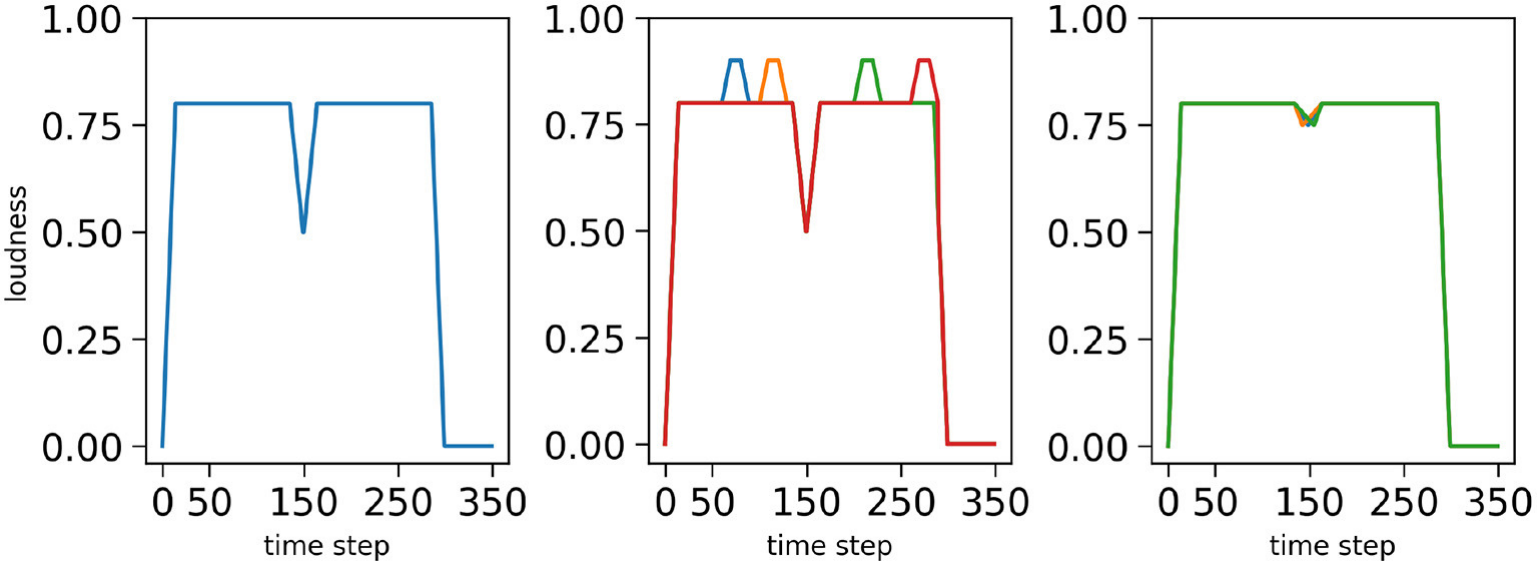
Loudness profiles for the bi-syllabic word “*pata*” used in the three simulation conditions: the nominal condition **(left)**, the “noisy-event” condition **(middle)** and the “hypo-articulation-event” condition **(right)**. Simulated time is on *x*-axes, normalized loudness (arbitrary units) is on *y*-axes. Degraded conditions **(middle, right)**, respectively correspond to the first noise level with one spurious event in a random position in the “noisy-event” condition, and to a dip value at 0.75 with random dip shapes in the “hypo-articulation-event” condition. The different colors correspond to different random degradations.

First, we consider a “nominal condition”, in which the stimulus presents no difficulty, that is to say, the stimuli loudness profiles are “smooth and regular,” such as shown in Figure 3. Second, to assess the model in more difficult situations, we define degraded versions of the loudness profiles, in two possible ways. In the first case, we add noise-events to the loudness, randomly positioned in portions where loudness is sustained in the nominal case: this may lead to detection of spurious loudness events by sensory processing (“noisy-event condition”). In the second case, we decrease the depth of the loudness dip found at syllable boundaries and randomly modify the shape of the loudness dip: this may lead sensory processing to miss syllabic onsets from the loudness signal (“hypo-articulation-event condition”). The three conditions, and the corresponding loudness profiles employed, are illustrated Figure 5 for the bi-syllabic word “*pata*.”

### 3.6. Simulation Configuration

We performed a set of simulations to evaluate the performance of the COSMO-Onset model. To do so, we simulate word recognition by the different model variants, for different words and for the various test conditions; for the test conditions that simulate a degradation of the stimulus, we applied different severity levels of the degradation. We now detail each of these components of our simulation set.

To recall, there are three considered variants of the model, in which syllable onset events are either assessed from bottom-up sensory information only (the “BU-only” model, in the following), or with top-down onset prediction combined with the *AND* operator (*AND* model), or, finally, with top-down onset prediction combined with the *OR* operator (*OR* model). The stimuli we used for the experiment are all non-monosyllabic words from the lexicon (21 different words out of the 28 in the lexicon, see Table 2). Monosyllabic words were not used as stimuli since they would only contain a single onset event, at the initial iterations; nevertheless, they are part of the lexicon and are evaluated as possible candidates by the model during word recognition. Each of these words is presented once to the three variant models in nominal test conditions (i.e., with nominal loudness profiles).

In the “noisy-event” test condition, we considered 5 possible severity levels, by varying the number of noise events applied to the loudness profile, from 0 (identical to the nominal case) to 4. Each noise event lasts 10% of the duration of the word, and its position is randomly drawn in the loudness profile of the word, ensuring that, when there are several noise events, they do not overlap (see examples of severity level 1 on Figure 5 middle).

In the “hypo-articulation” test condition, we considered 5 possible severity levels, by varying the depth of the loudness dip between syllables. In this dip, loudness decreases to a varying minimal value, from 0.6 (identical to the nominal case) to 0.8 (in which case the loudness dip between syllables is entirely removed, since loudness is at 0.8 inside syllables). The 5 possible values therefore are 0.6, 0.65, 0.7, 0.75 and 0.8. To introduce some variability, we randomly draw the precise time iteration, during the loudness dip, at which the minimal value is attained (except, of course, for perturbation level 0.8, since the dip is removed altogether. See examples of dip position at 0.75 on Figure 5 right).

Note that, while severity level 0 of the “noisy-event” test condition perfectly corresponds to the nominal case (and the simulations are thus not repeated), this is not the case for severity level 0 of the “hypo-articulation” test condition, since the time instant of the loudness minimal value is varied, which may affect onset detection. Whenever perturbations would be randomly generated, we performed 10 independent simulations for that condition. Overall, we therefore performed 21*3*(1 + 4*10 + 4*10 + 1) = 5,166 word recognition simulations: 21 word stimuli, 3 model variants, 1 for the nominal condition, 4*10 for the noisy-event condition, 4*10+1 for the hypo-articulation condition.

### 3.7. Performance Measures

In order to evaluate the performance of the model variants during the simulations of word recognition, we use two performance measures: correct recognition probability, and correct onset detection. First, correct recognition probability is measured as the probability ascribed by the model to the input word, at the last simulated iteration. The second performance measure aims at evaluating whether onset events were correctly detected by the model. As in any signal detection task, errors can be of two types: the model can incorrectly detect an onset where there was none, or the model can fail to detect an onset event in the stimulus. We therefore apply the F-score measure (Chinchor, 1992; Sasaki, 2007) to assess event detection performance. We compute *F* as:

$$F=2\frac{PR}{P+R}$$

with *P* the precision and *R* the recall. This scalar measure is therefore a trade-off between precision and recall; its value is high when errors, whatever their type, are few. In practice, we consider an event to be correctly predicted if the model generated an onset event internally in a 30-iteration wide time-window around the onset position in the stimulus (15 iterations before, 15 iterations after).

## 4. Results

We now report simulation results, to assess performance of the three model variants in the three experimental conditions: the “nominal” condition, the “noisy-event” condition, and the “hypo-articulation-event” condition. First, we detail an illustrative example, allowing to investigate the mathematical behavior of the model. This illustrative example is based on the input word “*pata”* in the nominal condition. Second, for each degradation condition, we first show the model behavior for the same input word “*pata,”* to illustrate mechanisms, before proceeding to systematic evaluation of performance over the whole simulation set.

### 4.1. Illustrative Example in Nominal Condition

**Figure 6:**
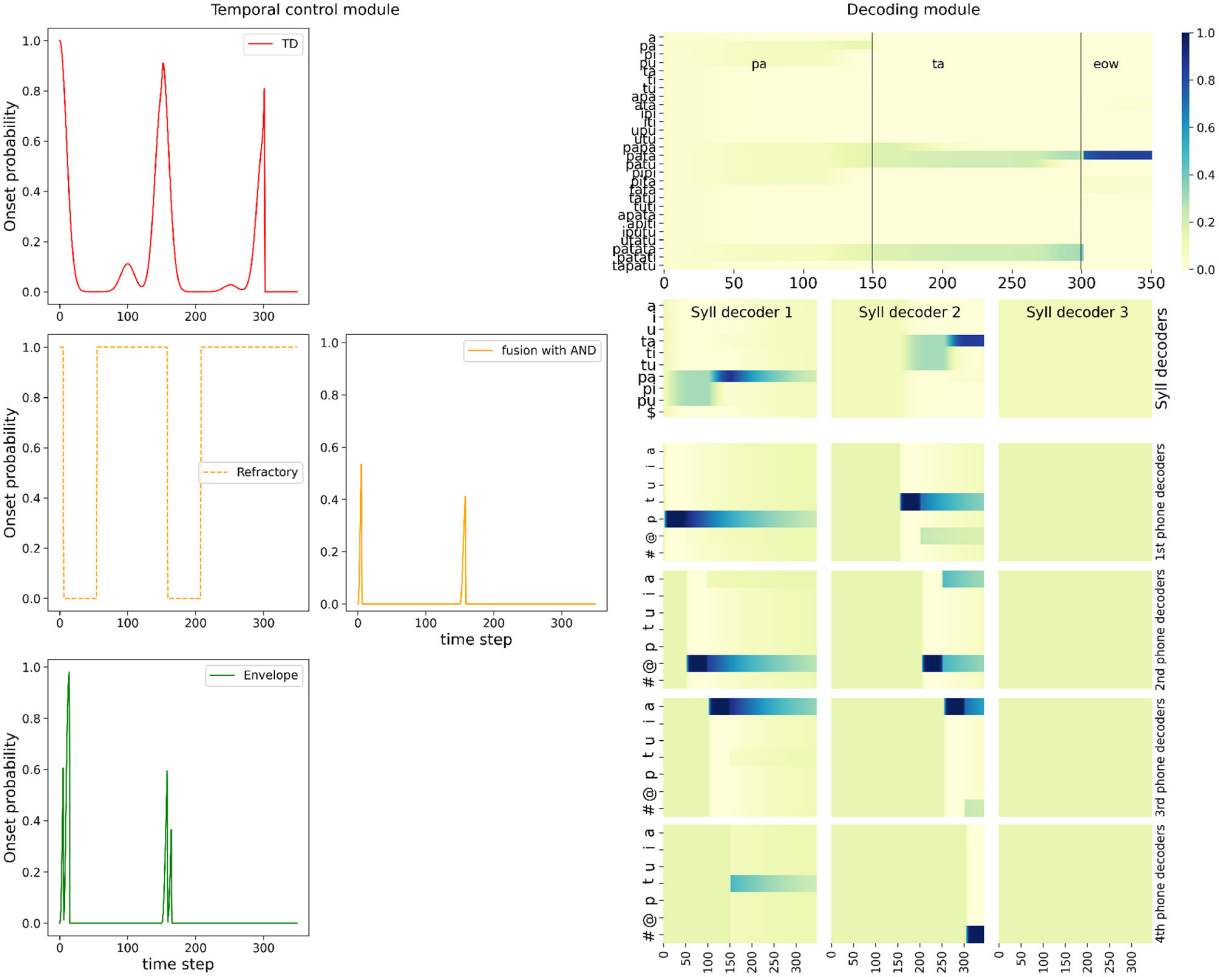
Example of simulation of the full model with the *AND* fusion in the nominal condition, on input word “*pata.”* Plots are organized to roughly map with corresponding positions in the model schema, see Figure 1. (**Left**, “Temporal control module” panel) Plot of the onset detection probabilities computed in the model (*y*-axis) as a function of simulated time (*x*-axis). Left column: In red, top-down onset prediction, in dashed orange, the probability of an onset being outside a refractory period, in green, onset probability based on sensory processing of stimulus loudness. **(Middle)**: in orange, the onset probability of the *AND* fusion model (**Right**, “Decoding module” panel) Top plot shows the probability (color coded, see the color bar on the right) over words (*y*-axis) as a function of time (*x*-axis). The vertical black bars, and annotations at the top of the plot recall the stimulus structure; in this example, the stimulus is the word “*pata,”* with the acoustic signal of the first syllable during the first 150 iterations, the one of the second syllable during the next 150 iterations, followed by silence. Second row: plots of the probabilities (color coded) over syllables (*y*-axis), as a function of time (*x*-axis) during the activation of the corresponding syllable decoder. Bottom four rows: plots of the probabilities (color coded) over phones (*y*-axis), as a function of time (*x*-axis). Plots for phone decoders are sorted vertically, with the first phone above and the fourth at the bottom.

Figure 6 shows the simulation of the full model with the *AND* fusion in the nominal condition, for the example stimulus word “*pata*.” It shows probability distributions computed by the model. Figure 6 left shows the different onset probability distributions in the temporal control module, and their evolution over time: the top-down onset prediction, the bottom-up onset detection composed of the refractory period and sensory event detection, and finally, the combined result with the *AND* fusion model. Figure 6 right shows probability distributions in the decoding module, with probability distributions over words (which provide the final output of the model), over syllables, and over phones.

Bottom-up onset detection shows that the model, based on sensory processing of the loudness envelope alone, would detect 2 events (Figure 6 left, bottom green curve), respectively around iterations 0, and 150. These, indeed, correspond to increase in the loudness profile for the stimulus word “*pata*” (see Figure 5 left). Since these are outside the refractory period (dashed orange curve, left column, middle row of Figure 5), these two onset events are maintained and “output” by the bottom-up branch of the temporal control module.

Top-down lexical knowledge would predict 3 onset events (Figure 6 left, top red curve), respectively around iterations 0, 150 and 300. The first two match with bottom-up onset detection, and, since we illustrate here the *AND* fusion model, they are maintained in the output of the temporal control module (orange curve in the middle of Figure 6). The third onset predicted by top-down knowledge is due to the fact that the presented stimulus, the word “*pata*” is a prefix of other words in the lexicon (tri-syllabic words “*patata*” and “*patati*”). At this stage of word recognition, these three words are equally probable (see Figure 6 top right plot), so that a third onset is likely. In this example, it is not confirmed by the bottom-up sensory event detection, and the *AND* fusion model filters it out.

At each detected onset, the model activates a new syllable decoder, so we observe that 2 syllabic decoders are involved in the model (Figure 6 bottom right portion). In each syllable decoder, the probability distributions over syllables evolve as acoustic input is processed, and the probability value of the correct syllable, that is, the one in the input, converges toward high values. We thus observe that each syllable decoder recognizes the appropriate syllable, which are /pa/ for the first syllable, and /ta/ for the second one. In the first syllable decoder, we observe a perfect competition for the first 100 time steps between all syllables beginning with phone *p*, which gets disambiguated when phone *a* is processed. The second syllable decoder is activated around iteration 150 (it is a uniform probability distribution before activation), and shows a similar dynamic: first, competition between all syllables starting with phone *t*, then recognition of the correct syllable /ta/. The third syllable decoder is never activated, and thus remains uniform during the whole simulation.

Within every syllable decoder, phonetic decoders get activated sequentially (Figure 6 bottom 12 plots of right portion). We observe behavior similar to the syllable decoders, except at a smaller timescale. Phone decoders stay uniform until their activation (this is especially visible for the phone decoders of the third syllable, which are never activated), then they decode the input, yielding, in this simulation, correct phone recognition, and after another onset is detected and predicted, the probability distributions gradually decay (this is especially visible for the phone decoders in the first syllable).

The probability distributions over syllables are then used, in the rest of the probabilistic computations in the model, to infer the probability distribution over words (Figure 6, top of the decoding module panel). Since syllable parsing was successful, so that syllable decoding was, too, then word recognition proceeds as expected, to recognize the word according to its syllables. Indeed, we observe that, at time step 150, that is, after decoding the first syllable /pa/, all words of the lexicon that start with /pa/ are equally probable. At time step 300, the lexical competition continues, and three words remain equally probable: the correct word “*pata*,” and two competitors, the words “*patata*” and “*patati*,” which embed the word “*pata*.” This issue has been discussed in the literature (Davis et al., 1998, 2002); in the current illustrative simulations we do not address this general question, as it is naturally solved since we only consider isolated words: after a few iterations in which the acoustic input represents silence, the recognized word is the correct one, the word “*pata*.”

We therefore observe correct onset detection (thus correct syllable parsing), but also correct phone, syllable and word recognition by the full model with *AND* fusion. Simulating the model in either the “BU-Only” or the *OR* fusion variant, in the nominal condition, also provides correct answers and thus, good performance (simulations not shown here, see below for model performance evaluation), with the exception of the activation of a third syllabic decoder, when the top-down model relies on the *OR* model, because the word “*pata*” is a prefix of other words in the lexicon (this is not shown here but can be observed in the final simulation, in the “hypo-articulation-event” condition: see **Figure 9**).

### 4.2. Noisy-Event Condition

A first challenge for the listener is when the acoustic signal is perturbed, because for instance of external noisy conditions. In that case, the speech envelope can be degraded, introducing extraneous fluctuations of loudness leading to detecting spurious events in the sensory processing of loudness. In other words, such spurious onsets would be detected by the bottom-up onset mechanism. Therefore, in this second simulation, we expect the “BU-only” model to result in erroneous syllable parsing, leading to incorrect syllable and word recognition. On the other hand, the complete model would rely on top-down lexical predictions of onset to “filter out” the unexpectedly detected onset (with the *AND* operator), leading to correct parsing and recognition.

**Figure 7:**
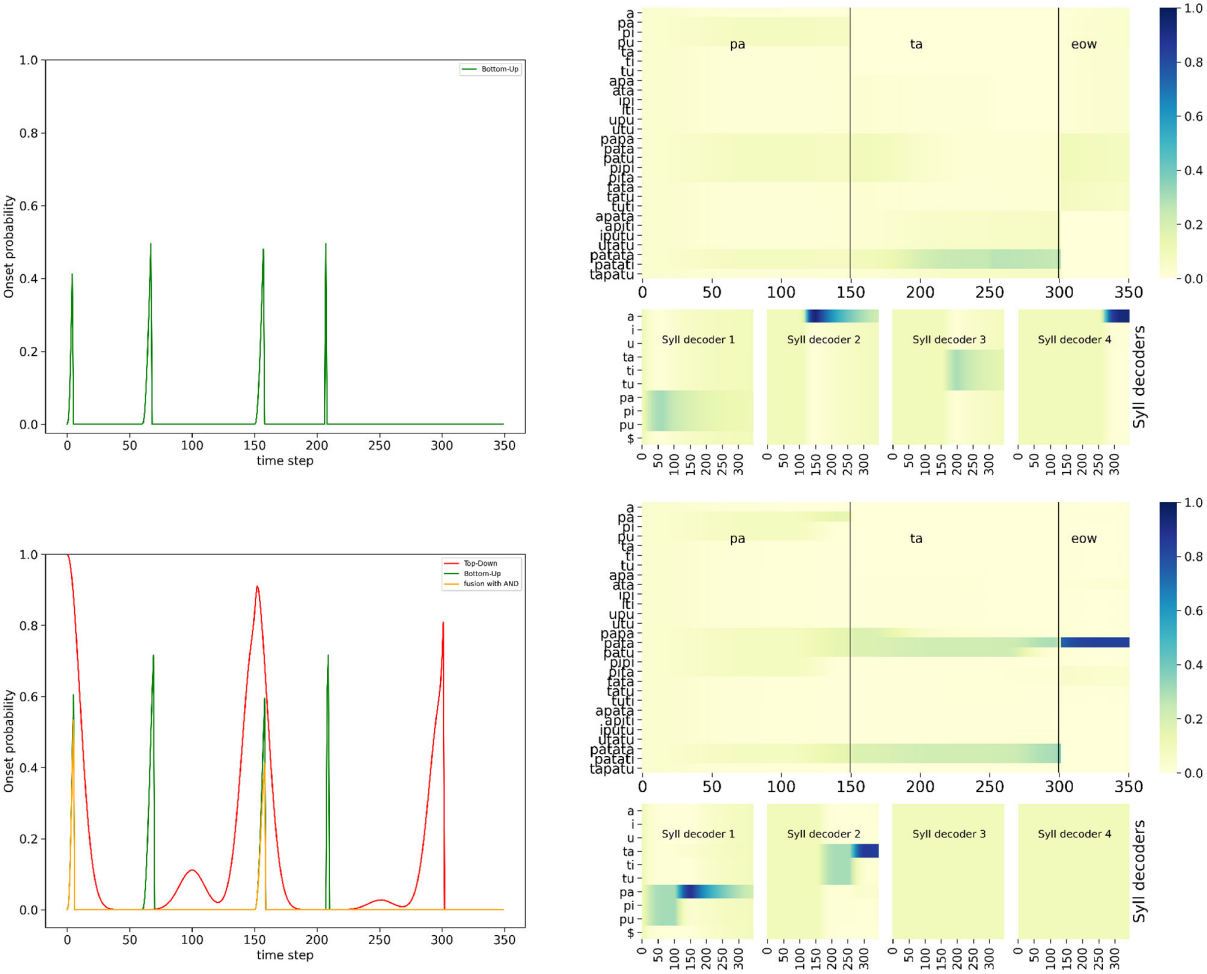
Example of simulation of the “BU-only” variant **(top row)** and the full model, with the *AND* fusion model **(bottom row)**, in the noisy-event condition, on input word “*pata.”* **(Left column)**: plots of onset detection probabilities; **(Right column)**: plots of word probabilities in the word decoder **(top row)** and syllable probabilities of the syllable decoders **(bottom row)**. Graphical content is presented in the same manner as in Figure 6 (except that onset probabilities are superposed in a single plot and the phone decoders are not shown).

Figure 7 shows the simulation of the “BU-only” variant of the model and of the full model (with the *AND* fusion model), on input word “*pata,”* with a degraded loudness profile that includes 2 spurious noise events. The simulation we selected here for illustration adds these events at iterations 60 and 200 (see Figure 5 middle). We observe that with the bottom-up onset mechanism alone (Figure 7 top row), the bottom-up onset mechanism “fires” 4 events, corresponding to the 4 energy rises in the loudness profiles: near the start, then at iterations 60, 150, and 200. These are well outside the refractory period of 50 ms, which would have otherwise filtered out these spurious onset events. Therefore, the bottom-up portion of the model detects 4 onset events. It leads to premature onset detection, which has a number of deleterious effects. First, it prematurely “closes” the first syllabic decoder, which was only fed with phone *p*, so that it is unable to correctly identify the first syllable in the input. Instead, the first syllabic decoder remains in an unresolved state of competition between all syllables that start with consonant phone *p*. Second, it prematurely opens the second syllabic decoder, that interprets the *a* vowel phone in the input as the syllable /a/, even though it is not legal in our lexicon in non-initial positions. This does not help resolving competition at the word level. Third, it correctly detects the real onset at time step 150, and opens a third syllable decoder supposed to decode the second syllable starting with phone *t*. But this is misaligned with the structure of the word “*pata*” which is bi-syllabic. Finally, the third decoder is prematurely closed, by the detection of the spurious onset event, near iteration 200. Overall, from one spurious event to another, the error in syllable parsing persists during decoding, and the BU-only variant is unable to correctly recognize the input word.

Compare with the simulation of the full model, with the *AND* fusion model, on the same stimulus (Figure 7 bottom row). We observe that, while the bottom-up onset detection mechanism would lead to propose an onset near time step 60, the top-down temporal prediction model does not confirm this proposal. Therefore, the *AND* fusion model results in filtering out this event. This also happens with the other spurious event near time step 200. Therefore, with the *AND* fusion model, only the two “real” onsets are detected, that is to say, the ones at the start of each syllable. As a consequence, the behavior of the *AND* fusion model in the “noisy-event” condition is quite the same as in the nominal condition, with correct syllabic parsing, phone recognition, syllable recognition and word recognition.

**Figure 8:**
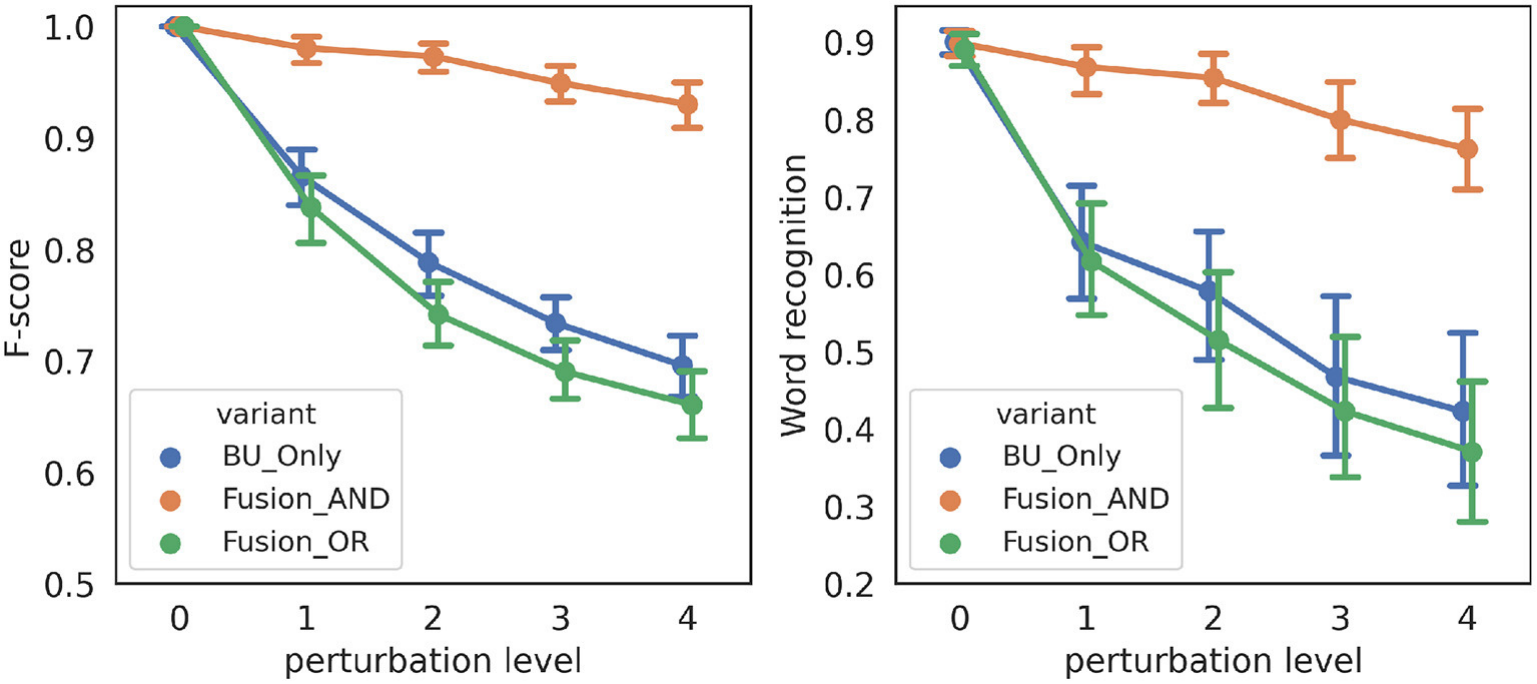
Performance of the three variant models in the “noisy-event” condition. **(Left)**: F-score (*y*-axis) as a function of the severity of degradation (*x*-axis). **(Right)**: word recognition probability (*y*-axis) as a function of the severity of degradation (*x*-axis). Every data point is averaged, over 21 words, and, where applicable, over 10 independent simulations with different randomly drawn perturbations.

Figure 8 shows performance measures for the three variant models in the “noisy-event” condition, across all simulations. We first observe that both performance measures are highly correlated, suggesting that correct event detection relates with correct word recognition. Second, when there is no perturbation (perturbation level 0), all variant models have the same performance, which is expected since top-down event prediction is redundant in this case with events that can be detected from the input signal. Third and finally, we also observe that the higher the severity level of degradation, the more performance decreases. Indeed, as degradation increases, the chance of having noise perturbations outside refractory periods increases, thus leading to more chance for spurious onset events. However, we observe that the model with the *AND* fusion is the most robust, as its performance decreases less with perturbation.

### 4.3. Hypo-Articulation-Event Condition

In the second challenge we consider, degradation of the loudness profile leads to “removing out” onset events, for instance with an external perturbation masking a dip in acoustic energy at the syllabic boundary, or with this dip being much smaller, maybe because of hypo-articulation, or an error in speech planning, or excessive speed in speech articulation leading to speech slurring, etc. In that condition, we expect the “BU-only” variant of the model to miss onset events, leading to incorrect syllabic parsing, thus incorrect recognition. On the other hand, the complete model, with the *OR* operator, would use the lexically predicted onsets to insert them where the sensory onsets were missed, leading to correct parsing and recognition.

**Figure 9:**
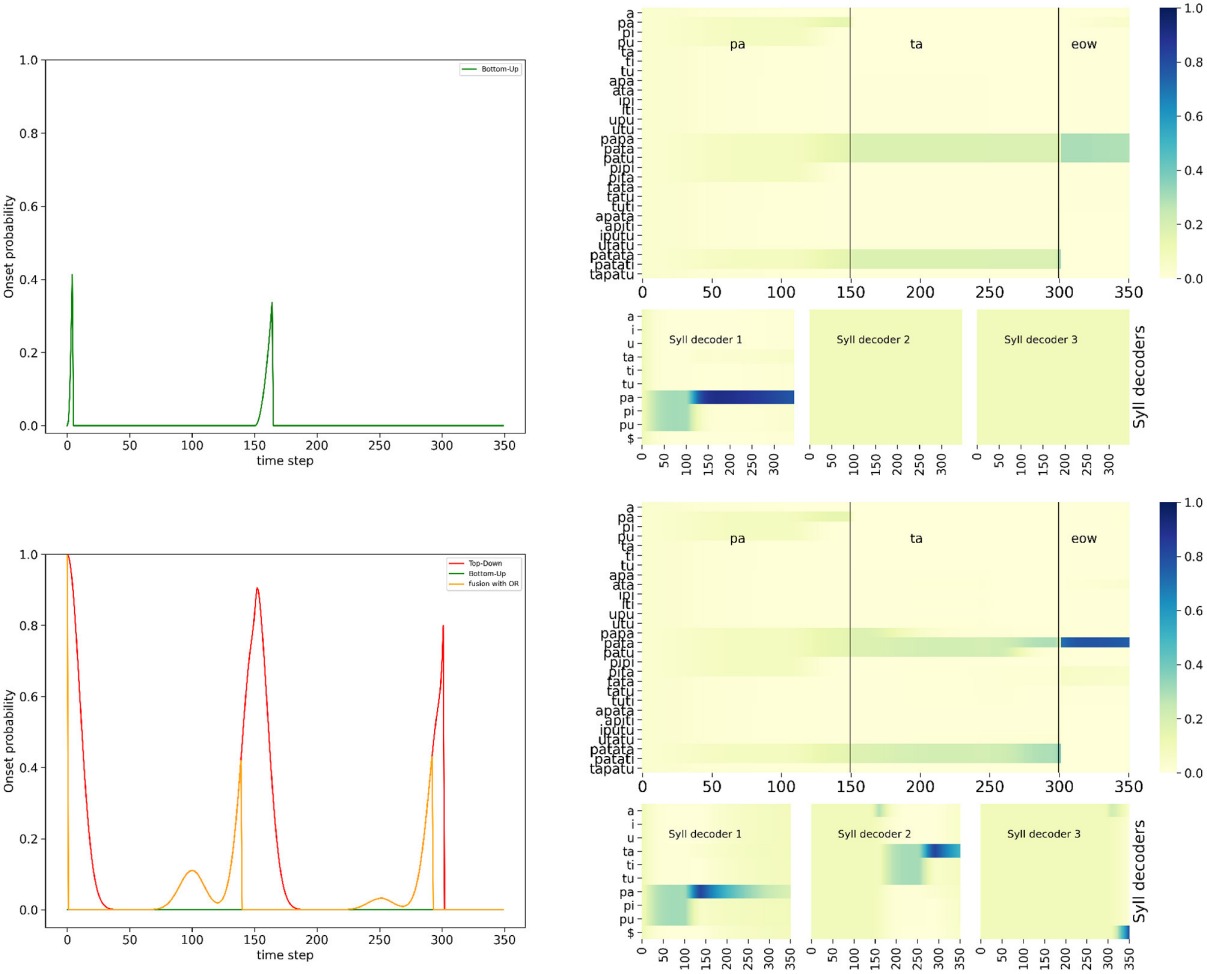
Example of simulation of the “BU-only” variant **(top row)** and the full model, with the *OR* fusion model **(bottom row)**, in the hypo-articulation-event condition, on input word “*pata.”* Graphical content is presented in the same manner as in Figure 7.

Figure 9 shows the simulation of the “BU-only” variant of the model and of the full model (with the *OR* fusion model), on input word “*pata,”* with the degraded loudness profile that decreases the dip depth in acoustic loudness at the syllabic boundary (see Figure 5 right). We observe that the “BU-only” variant of the model does not ascribe a probability for the second onset prediction that is high enough (the probability is lower than the decision threshold at 0.4), and therefore it misses the second onset (near time step 150), so that the first syllabic decoder stays activated for too long. Although it correctly recognizes the initial /pa/ syllable, it never activates the second syllable decoder. This leads to unresolved competition at the word level between all the bi-syllabic words starting with syllable /pa/, and, ultimately, incorrect word recognition.

In contrast, with the *OR* fusion model (Figure 9), the top-down onset prediction allows to recover the missed onset event at time step 150, which helps to avoid the problem of faulty syllabic parsing and misalignment of the second syllabic decoder with the stimulus. In this condition, the full model with the *OR* fusion model leads to correct syllabic parsing, phone recognition, syllable recognition and word recognition. Notice, however, that simulations here are not exactly the same as those of the model in the nominal condition, with two differences that merit attention.

Indeed, we first observe that the syllabic decoders are a few iterations ahead the stimulus: for instance, whereas the syllabic boundary between the first and second syllables is exactly at time step 150 in the stimulus, the first onset event resulting from the *OR* fusion model is around time step 140. This leads the second syllabic decoder to process, for a few iterations, the end of the first phone *a* in stimulus “*pata.”* This slight temporal misalignment is due to the value we set for the onset decision criterion, at 0.4. Such a value is reached early of the “bump” in onset probability provided by the lexical model, that correctly peaks at time step 150 [Figure 9 bottom left plot, compare the lexical prediction (red curve) and output of the *OR* fusion model (orange curve)].

The second notable behavior in this simulation is the activation of a third syllabic decoder. Indeed, the lexical onset prediction model is aware of words in the lexicon which embed “*pata*.” Therefore, up to time step 300, there is an unresolved competition, at the word recognition level, between the embedding words containing “*pata*” and “*pata*” itself. A third syllable could then, from the lexical prediction, be expected, so that an onset event is lexically generated. This leads to activating a third syllable decoder, which mostly processes the “end of word” marker in the acoustic input (after the few iterations where it processes the end of the second *a*, because of the slight temporal misalignment discussed above). Observing a third syllable “composed of silence” is only consistent with the word “*pata”* in the lexicon, so that it is, ultimately, correctly recognized.

**Figure 10:**
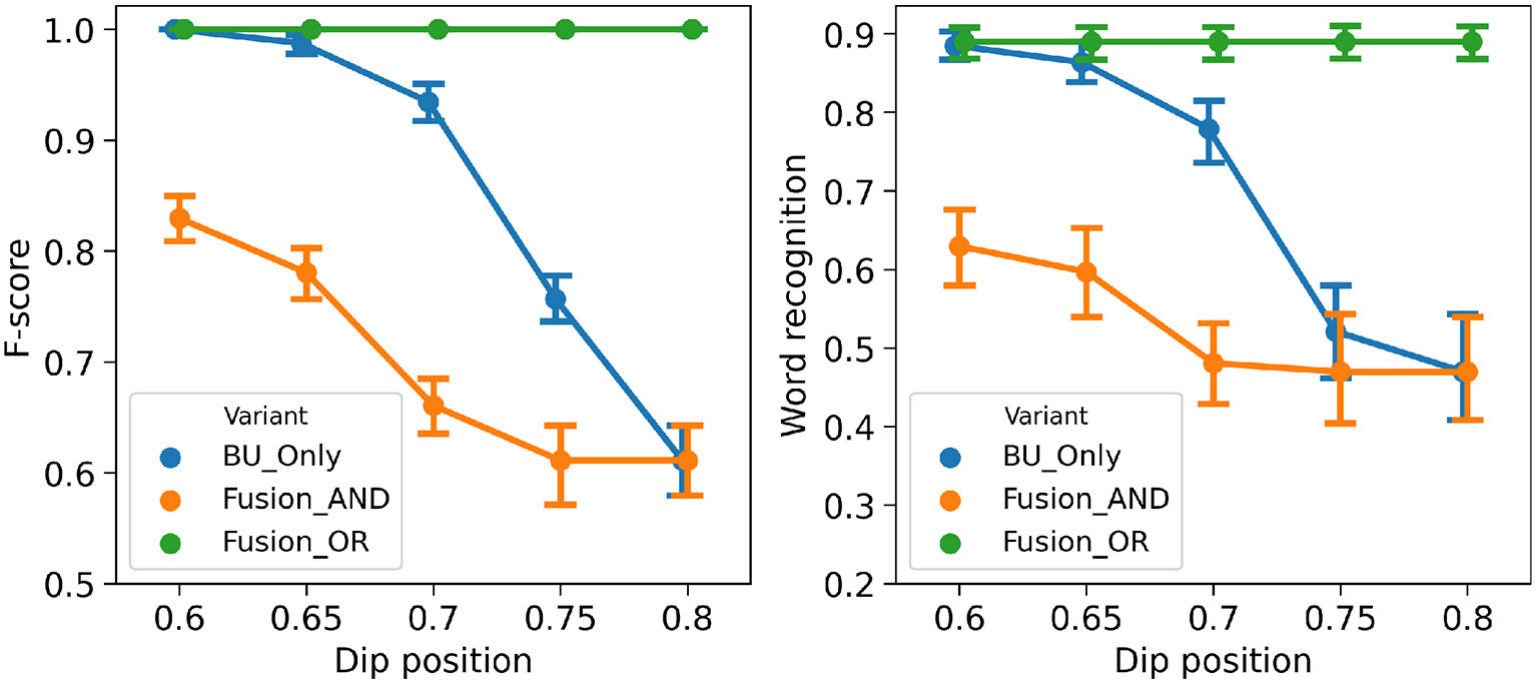
Performance of the three variant models in the “hypo-articulation-event” condition. **(Left)**: F-score (*y*-axis) as a function of the severity of degradation (*x*-axis). **(Right)**: word recognition probability (*y*-axis) as a function of the severity of degradation (*x*-axis). Severity of degradation is measured with the minimal value attained in the loudness dip at syllable boundaries (“dip position” in plot labels; 0.6 corresponds to a well-marked dip, 0.8 removes the loudness dip altogether). Every data point is averaged, over 21 words, and over 10 independent simulations with different randomly drawn perturbations.

Figure 10 shows performance measures for the three variant models in the “hypo-articulation-event” condition, across all simulations. First, we observe, here again, that both performance measure correlate. Second, we observe that, contrary to simulations in the “noisy-event” condition, all three model variants do not have the same performance for the less degraded condition. Indeed, in our simulation, we randomly select the time iteration at which the minimal value is reached; this changes the geometry of the dip in loudness, so that, even though it has nominal depth (when dip position is 0.6), it can mis-align onset detection and prediction, which negatively affects the *AND* fusion model. Third and finally, we also observe that performance decreases as degradation increases, for the “BU-only” and the *AND* fusion models. The performance of the *OR* fusion model, on the contrary, does not decrease as perturbation increases, indicating robustness of the *OR* fusion model in the “hypo-articulation-event” condition.

## 5. Discussion

In this paper, we explored the processes of syllabic segmentation, with a specific focus on the potential role of top-down temporal predictions. Simulations show that, even though, in ideal conditions, acoustic envelope processing alone may be sufficient for efficient syllabic segmentation, in adverse conditions, the modulation of bottom-up processes by top-down knowledge improves segmentation of speech into its syllabic constituents. Two sets of adverse conditions were tested. The first set corresponds to the case where the bottom-up onset mechanism detects extraneous events due to noisy acoustic signal. The second one corresponds to the case where the speech envelope is degraded, possibly in certain phonetic contexts or with hypo-articulated speech, so as to miss crucial events for segmentation. In these two cases, the “BU-only” variant of the model performs poorly, segmentation of the speech input into constituent units is negatively impacted, and hence the word recognition process is impaired.

To evaluate how such an impaired performance can be mitigated by integrating top-down lexical knowledge providing temporal predictions about syllabic onsets, we proposed two fusion models for combining the bottom-up detection and top-down prediction submodels. The first one aims at compensating for the case where the bottom-up branch detects spurious onsets, requiring the top-down branch to filter out these spurious events. This is dealt with by the *AND* fusion model, which decides that there is an onset only if the probability distributions in both submodels are coherent, enabling the top-down branch to inhibit the detection of spurious events in the bottom-up one. The second fusion model corresponds to the case where the bottom-up branch misses important events. This is dealt with by the *OR* fusion model, which detects an onset when at least one of the two branches “fires,” enabling the top-down branch to recover the events missed by the bottom-up one. Altogether, this confirms that appropriate fusion in the full model allows to mitigate the weak performance of the “BU-Only” variant of the model.

Therefore, the main goal of the present study, which was to design and test fusion models associating bottom-up acoustic envelope processing and top-down timing predictions from higher linguistic levels, is reached, and this provides the major contribution of this paper. We now have at our disposal a general Bayesian architecture associating temporal control and phonetic decoding, that can be implemented, tuned or modified in various ways, tested on speech stimuli and possibly used for generating predictions for future neurocognitive experiments. Of course, the simulations we proposed here are preliminary, and should be extended in the future, in various directions. We discuss this below.

###  From “Toy” Stimuli to Realistic Speech Processing

First of all, it is important to acknowledge that the current material used as input to the model is far from real speech. To be able to finely monitor the model output at this preliminary stage, we designed toy stimuli. Specifically, the spectral description of the acoustic stimulus was limited to the first two formants. The first layer in COSMO-Onset, that is the Phone Sensory Layer, currently takes for granted the feature extraction from the speech input by directly implementing phone recognition from the first two formants, while realistic spectral analysis of speech utterances would rather exploit a bank of auditory filters (e.g., gammatones Patterson et al., 1992; Hohmann, 2002 or mel-cepstrum analysis Rabiner, 1989). This is also the case for the synthetic loudness curves used to simulate the speech envelope and the two kinds of adverse perturbations applied to these curves, together with the simplified loudness processing in the bottom-up branch performing a “simplistic” bottom-up onset detection with straightforward envelope analysis. A further step in the development of COSMO-Onset will be to consider more realistic neuro-computational models able to track the signal envelope and adapt in an online manner to variations in instantaneous syllabic frequency, as in oscillatory models such as the ones developed by Hovsepyan et al. (2020), Hyafil et al. (2015), and Räsänen et al. (2018) (see also the neuro-physiological refinements recently introduced by Pittman-Polletta et al., 2021). Importantly, these various existing models should help provide COSMO-Onset with a possible neurophysiological implementation of the temporal processing component of the algorithmic structure presented on Figure 1, which would make the relationships between the present simulations and real neurophysiological data more straightforward.

###  Efficiently Combining Bottom-Up and Top-Down Information for Syllabic Parsing

The simulations presented in section Results suggest a rather clear overall picture. Firstly, in the “hypo-articulation simulation set,” generating missing events in the bottom-up branch, the *OR* model behaves efficiently and outperforms the “BU-Only” model in terms of both detection accuracy and recognition score. Secondly, in the “noisy-simulation set,” generating spurious events, the *AND* model discards most of these spurious events and outperforms the “BU-Only” model once again in terms of both detection accuracy and recognition score. Notice that in both cases, the bottom-up branch performs actually better than the non-adapted fusion model. Indeed, the *AND* model degrades event detection when it is already difficult in the hypo-articulation case, probably because of a slight asynchrony between the bottom-up and the top-down branches; and the *OR* model slightly increases the number of inaccurate or spurious events detected in the noisy case, probably because the top-down information enhances spurious envelope modulations.

Globally, this raises the question of selecting the right model for the right stimulus condition. This falls into the general question of model selection and averaging, for which literature is abundant (e.g., Wasserman, 2000; Burnham and Anderson, 2004). This would suggest various ways of analyzing the probabilistic content of each of the three models “BU-Only,” *AND* and *OR* and selecting or averaging their output accordingly. Importantly, the rationale of the two sets of simulations suggests that some exogenous contextual criterion could be used for model selection. Thus, if the system is able to extract some evaluation of the level of noise or the quality of articulation during a short period of time, this information could be used as a proxy to select the *AND* or the *OR* fusion model accordingly, or even to combine them. The same kind of endogenous or exogenous information could also be used as a prior or a weight in the Bayesian fusion process involved in both the *AND* and the *OR* model. For example, instantaneous estimates of the noise level could act as a weighing factor in the *AND* Bayesian fusion process, increasing/decreasing the respective roles of the bottom-up and top-down branches accordingly. The Bayesian framework that we have adopted all along this work in the development of the COSMO-Onset model is obviously adapted to study and explore all the corresponding questions about model selection and fusion.

Finally, if there indeed exist two different fusion modes, namely an *AND* and an *OR* behavior, this raises some interesting questions for cognitive neurosciences, asking whether specific neural markers could be associated to a shift from one mode to the other. Indeed, it has been proposed, for instance by Arnal and Giraud (2012) and Giraud and Poeppel (2012), that there could exist specific frequency channels, respectively, associated to bottom-up (theta channel) and top-down (beta channel) messages. The shift from the *AND* to the *OR* behavior, possibly associated to noisy conditions vs. hypo-articulation, would result in different coordination in time between theta and beta bursts, that could be explored in adequate neurophysiological paradigms.

###  Relation With Classical Models in Psycholinguistics

The decoding module in COSMO-Onset has a hierarchical structure similar to classical psycholinguistic models like TRACE (McClelland and Elman, 1986), though it replaces neural computations in TRACE by probabilistic computations. Still, a model like TRACE incorporates feedback from higher to lower levels in the decoding process, which means that not only the perception of phonemes influences the perception of words, but, conversely, word recognition influences phoneme identification, although the way this feedback process influences perception is still a matter of debate (Norris et al., 2016). We plan to include such feedback processes into the COSMO-Onset model, to provide top-down predictive processes at the decoding stage, in coherence with the predictive coding framework (Friston, 2005; Friston and Kiebel, 2009). This, combined with the architecture we proposed in the COSMO-Onset model, raises an interesting issue. Indeed, the temporal control module of COSMO-Onset can be seen as an “alternate” pathway of the decoding module for information propagation into the model. In other words, the COSMO-Onset model could yield specific predictions about the mathematical influence of syllabic parsing on lexical access; these could be compared with psycholinguistic data on spoken word recognition.

###  Assessment in Relation With Experimental Data on Speech Perception

Finally, an important perspective concerns the ability of a model like COSMO-Onset to deal with specific experimental conditions in which the role of top-down processes in syllabic parsing and onset detection could be crucial. We have particularly in mind two types of experimental paradigms related to transformations of the temporal structure of speech.

A first challenge concerns the processing of temporally compressed data. Ghitza and Greenberg (2009) showed that speech perception resists rather well to temporal compression, up to a factor of 3, where intelligibility is degraded, and that the introduction of silences at specific moments provides the listener with “additional decoding time,” which restores intelligibility. This first paradigm provides a good test-bed for assessing the resilience of the bottom-up temporal control module in word processing. In its present stage, the COSMO-Onset model is not likely to be able to simulate this behavioral pattern in a realistic manner. Indeed, this would probably require an intrinsic temporal scale, to adapt to the stimulus speech rate, which would set limits for compression, likely related to limits of an oscillatory process. Hence, this first set of experimental data will probably require to replace the oversimplified onset detection algorithm based on loudness increase in the present version by a real neurocomputational oscillatory model such as the ones by Hyafil et al. (2015) and Hovsepyan et al. (2020).

A second challenge concerns the already mentioned study by Aubanel and Schwartz (2020) showing that natural speech is more intelligible in noise than speech rendered isochronous, while isochrony also plays a role in helping intelligibility, but to a lesser extent. Naturalness and isochrony play here complementary roles which could fit quite well with the existence of a bottom-up onset detection branch exploiting isochrony, and a top-down prediction branch exploiting naturalness. Once again, the beneficial role of isochrony would probably require an oscillatory process in bottom-up onset detection. However, the role of naturalness is incompatible at this stage with the existing neurocomputational syllabic parsing models such as those of Ghitza (2011); Hyafil et al. (2015); Hovsepyan et al. (2020), since it requires top-down predictions about speech timing. Here, an experimental prediction is that a fusion model associating bottom-up detection with top-down predictions would hence be necessary. Since the experimental paradigm tested by Aubanel and Schwartz (2020) is based on perception in noise, it is likely that the *AND* fusion model would be required here. The experimental test-bed provided by this study could enable to refine the precise coordination in time between the top-down and the bottom-up detection processes. It could indeed be suggested that the top-down predictions from the statistics of natural speech timing could provide a window, probably rather large, where an event should be searched for. In such a window, the bottom-up branch could exploit a temporally narrow envelope processing algorithm to provide a final precise estimation of syllabic events, later used for word recognition and sentence comprehension.

## Data Availability Statement

The original contributions presented in the study are included in the article/Supplementary Material, further inquiries can be directed to the corresponding author/s.

## Author Contributions

MN, J-LS, and JD designed the model, experiments, and wrote the manuscript. MN implemented the model and simulations. All authors contributed to the article and approved the submitted version.

## Conflict of Interest

The authors declare that the research was conducted in the absence of any commercial or financial relationships that could be construed as a potential conflict of interest.

## Publisher's Note

All claims expressed in this article are solely those of the authors and do not necessarily represent those of their affiliated organizations, or those of the publisher, the editors and the reviewers. Any product that may be evaluated in this article, or claim that may be made by its manufacturer, is not guaranteed or endorsed by the publisher.
