# Supplementary material for: COSMO-Onset: A Neurally-Inspired Computational Model of Spoken Word Recognition, Combining Top-Down Prediction and Bottom-Up Detection of Syllabic Onsets
Source: Front Syst Neurosci. 2021 Aug 4;15:653975. doi: 10.3389/fnsys.2021.653975 (PMC8371689; doi:10.3389/fnsys.2021.653975)
Supplement: Supplementary file 1 [file Data_Sheet_1.PDF]

# Supplementary Material

## A FULL MODEL SPECIFICATION

To specify the full model (see Figure 1 in the main paper), we use the Bayesian Programming framework (Lebeltel et al., 2004; Bessière et al., 2013; Diard, 2015), which is a methodology for defining probabilistic models. In this methodology, a joint probability distribution is defined following three steps: first, all the relevant variables are listed and their domains are defined; second, the joint probability distribution is decomposed into a product of terms, and some of these are simplified thanks to conditional independence hypotheses; third and last, all terms of the decomposition need to be specified, and their parameters possibly identified from data using a learning mechanism. Once the joint probability distribution is fully defined in this manner, it can be used to "answer questions", that is to say, compute terms of interest by applying Bayesian inference. We now provide the complete definition of the model by following these four steps [1].

### A.1 Variables

In our methodology, probabilistic variables are defined by their name and domains. The way we specify variable names, in our notation, deserves introduction. Indeed, since the same representational space (e.g., the syllabic space) can be shared by several variables depending on their roles in the model (e.g., the perceived information or the lexically predicted information), then we compose variable names. For instance, $SyP$ would be the perceived syllable and $SyL$ would be the lexically predicted syllable. Furthermore, we use subscript indices to denote "position" in the speech sequence, and superscript indices to denote "time instants". For instance, $SyP_2^{50}$ would be the probabilistic variable representing knowledge that the models has, at time instant 50, about the second syllable perceived. Finally, we use a shorthand to denote sets of variables: $SyP_{1:N}^{1:T}$ is the set of all variables about perceived syllable variables, for all positions 1 to $N$, with $N = 3$ the number of syllable decoders and all time instants 1 to $T$, with $T$, arbitrarily set to $500$, which is the longest word duration in the lexicon. The different variables of the model are as follows.

- $I_{1:12}^{1:T}$ represent the spectral contents of the acoustic signal Input (in the following, we capitalize the part of the variable definition which motivates its name). They take continuous values in the 2 dimensional space representing the first two formants F1, F2 (in barks).
- $\Delta L_{1:12}^{1:T}$ represent the derivative of the Loudness of the acoustic signal. The loudness variable used to describe the stimulus, which are not represented inside the model, and thus have no probabilistic variables associated, take continuous values; therefore, it is also the case for the $\Delta L_{1:12}^{1:T}$ variables.
- $Sil_{1:12}^{1:T}$ are binary variables derived from the loudness of the stimulus. They indicate "Silence" instants in the input by locating places where the loudness is null (i.e., 1 represents a silent time step, 0 otherwise).
- $FeP_{1:12}^{0:T}$ represent the set of possible phones (*Fe* for "features"), which is a discrete set of values:

$$Fe = \{a, i, u, p, t, @, \#\} ,$$

  where $/@/$ represents transition phones (acoustic features outside of the other categories) and $/\#/$ is an end-of-sequence marker. $FeS_{1:12}^{1:T}$ and $FeL_{1:12}^{1:T}$ are defined over the same domain, but used for

[1] Note that there is a private Github with all the codes (model implementation and notebooks for simulations) available on demand. For the interested reader, please contact at mamady.nabe@univ-grenoble-alpes.fr

different portions of the model: *FeP* variables represent Perceived features, *FeS* represent Sensed features and *FeL* represent Lexically predicted features.

- $SyP^{0:T}_{1:3}$ represent the set of possible Syllables, which is a discrete set of values:

$$Sy = /a/, /i/, /u/, /pa/, /pi/, /pu/, /ta/, /ti/, /tu/ \ .$$

  $SyS^{1:T}_{1:3}$ and $SyL^{1:T}_{1:3}$ are defined over the same domain and, as previously, for different portions of the model: *SyP* variables for Perceived syllables, *SyS* for Sensed syllables and *SyL* for Lexically predicted syllables.
- $WP^{0:T}$ represent the set of possible Words, which is a discrete set of values. All the words of the lexicon can be found in Table 2 of the main paper. $WS^{1:T}$ are defined over the same domain, and, as above, *WP* variables represent Perceived words and *WS* variables represent Sensed words.
- To connect different portions of the model, a set of so-called "coherence variables" (Bessière et al., 2008; Gilet et al., 2011) are defined; they are binary variables, taking values 0 or 1. They are all represented graphically identically as "$\lambda$" nodes in Figure 1, but they actually have different mathematical notations. For instance, $\lambda FeSP^{1:T}_{1:12}$ connect the sensed and perceived phone variables, $\lambda FePL^{1:T}_{1:12}$ connect the perceived and lexically predicted phones, and so on and so forth for $\lambda SySP^{1:T}_{1:3}$, $\lambda SyPL^{1:T}_{1:12}$ and $\lambda WSP^{1:T}$.
- $A^{1:T}_{1:15}$ are sets of so-called "control variables" (Phénix, 2018; Ginestet et al., 2019), which are Boolean variables. They are used to control the amount of information transferred through the coherence variables between the different representational layers. There are 15 sets of such control variables: 3 of them, $A^{1:T}_{13:15}$, control the quantity of information between the syllable lexical and perceptual layers and the other 12, $A^{1:T}_{1:12}$, control the quantity of information transferred between the feature sensory and perceptual layers; this mechanism to control information transfer is used to activate the different phone and syllable decoders sequentially.
- $OTD^{1:T}$, $OBU^{1:T}$, $OREF^{1:T}$ and $OC^{1:T}$ are Boolean variables, to represent the probability that there is a syllabic Onset event. The *OTD* variables represent the prediction of syllable onset events derived from word lexical knowledge, in a "Top-Down" manner; *OBU* represent syllable onset events detected from acoustic envelope processing, in a "Bottom-Up" manner; *OREF* represent syllable onsets (more precisely, their absence thereof) during the REFractory period after the preceding onset; finally, *OC* represent the syllabic onsets resulting from the Combination of available information about these events (either from the *OBU* and *OREF* variables in the "BU-only" variant of the model, or from a fusion model with the *OTD* variables in the complete model).

## A.2 Decomposition

We now consider the joint space described by the conjunction of all the variables we defined above. The joint probability distribution (*JD*) cannot, of course, be defined directly; instead, we decompose it into a product of terms and simplify them with conditional independence assumptions. This results in a dependency structure, which is graphically represented in Figure 1. In other words, the conditional independence assumptions correspond to the structural choices that result in the overall architecture of the model. These structural choices are broadly motivated by theoretical frameworks of the architecture, for instance assuming a separation between the temporal control module and the decoding module. This assumption is shared with other models, such as the TEMPO model (Ghitza, 2011). Another example is the three-layer architecture separating phone, syllable and word levels, as a variant of the TRACE model (McClelland and Elman, 1986).

To implement these structural assumptions into the model, we decompose the joint probability distribution into:

$$
\begin{aligned}
JD &= P\left(\begin{array}{l}
WP^{0:T}\ WS^{1:T}\ SyL_{1:3}^{1:T}\ SyP_{1:3}^{0:T}\ SyS_{1:3}^{1:T}\ FeL_{1:12}^{1:T}\ FeP_{1:12}^{0:T}\ FeS_{1:12}^{1:T}\\
\lambda WSP^{1:T}\ \lambda SyPL^{1:T}\ \lambda SySP^{1:T}\ \lambda FeSP^{1:T}\ \lambda FePL^{1:T}\\
I_{1:12}^{1:T}\ \Delta L_{1:12}^{1:T}\ Sil_{1:12}^{1:T}\\
A_{1:15}^{1:T}\ OTD^{1:T}\ OBU^{1:T}\ OC^{1:T}\ OREF^{1:T}
\end{array}\right)\\
&= \left[\begin{array}{l}
P(WP^0)\times\prod_{i=1}^{3}P(SyP_i^0)\times\prod_{j=1}^{12}P(FeP_j^0)\\
\times\prod_{t=1}^{T}\left[\begin{array}{l}
P(A_{1:15}^t)\times P(OREF^t \mid A_{1:15}^t)\times P(OC^t \mid OTD^t OBU^t OREF^t)\times P(OTD^t \mid WS^t)\\
\times P(WP^t \mid WP^{t-1})\times P(\lambda WSP^t \mid WS^t\ WP^t)\times P(WS^t)\\
\times\prod_{i=1}^{3}\left[\begin{array}{l}
P(SyL_i^t \mid WS^t)\times P(\lambda SyPL_i^t \mid SyP_i^t\ SyL_i^t)\times P(SyP_i^t \mid SyP_i^{t-1})\\
\times P(\lambda SySP_i^t \mid SyS_i^t\ SyP_i^t\ A_i^t)\times P(SyS_i^t)\\
\times\prod_{j=4(i-1)}^{4i}\left[\begin{array}{l}
P(FeL_j^t \mid SyS_i^t)\times P(\lambda FePL_j^t \mid FeL_j^t\ FeP_j^t)\\
\times P(FeP_j^t \mid FeP_j^{t-1})\times P(\lambda FeSP_j^t \mid FeS_j^t\ FeP_j^t\ A_j^t)\\
\times P(I_j^t \mid FeS_j^t)\times P(FeS_j^t)\times P(\Delta L_j^t)\\
\times P(OBU^t \mid \Delta L_j^t)\times P(Sil_j^t \mid WS^t)
\end{array}\right]
\end{array}\right]
\end{array}\right]
\end{array}\right]
\end{aligned}
$$

Inside the temporal product ($\prod_{t=1}^{T}\ldots$), the first four terms , along with the $P(OBU^t \mid \Delta L_j^t)$ in the innermost product, relate to the temporal control module of the COSMO-Onset model, and the other terms relate to the decoding module. Terms related to the decoding module are organized “vertically” to match with the structure of the graph representing the decoding module of the model, in Figure 1 (from the *WP* variables at the top to the stimulus variables at the bottom).

## A.3 Parametric forms

We now define all the parametric forms of the probability distributions of the terms that appear in the decomposition of the joint probability distribution.

1. The prior probability distributions of the temporal perceptual models (that is to say, over *WP*, *SyP* and *FeP*), are all defined as discrete uniform probability distributions over their domains (resp., over words of the lexicon, syllables and phones): $\forall w, P([WP^0 = w]) = \frac{1}{|W|}$, $\forall s, i, P([SyP_i^0 = s]) = \frac{1}{|Sy|}$, $\forall f, j, P([FeP_j^0 = f]) = \frac{1}{|Fe|}$.
2. The dynamic probability distributions of the temporal perceptual models (that is to say, over *WP*, *SyP* and *FeP*), are all defined as discrete conditional probability distributions over their domains (resp., over words of the lexicon, syllables and phones). These are “quasi-Dirac” distributions, that is to say, they have almost probability 1 on their “diagonal”, and a residual, non-zero probability everywhere else. For instance, for the phone perceptual dynamic model we note:

$$
P([FeP_j^t = f^t] \mid [FeP_j^{t-1} = f^{t-1}]) = \begin{cases} \frac{1+leak_{Fe}}{1+|Fe|leak_{Fe}} & \text{if } f^t = f^{t-1},\\ \frac{leak_{Fe}}{1+|Fe|leak_{Fe}} & \text{otherwise,}\end{cases}
$$

with $leak_{Fe} = 10^{-3}$. The dynamic models over syllables and words are defined in a similar manner, with parameters $leak_{Sy} = leak_W = 10^{-3}$. We note that this value is set empirically here; in the

**Table S1.** Parameters of the Gaussian distributions over the spectral contents, in $F_1, F_2$ space, for the term $P(I_j^t \mid [FeS_j^t = f])$. For the vowels */a, i, u/*, the parameters are identified from the *VLAM* dataset (see Figure 2). Mean parameters are defined as vectors $\begin{pmatrix} F_1 & F_2 \end{pmatrix}$ and covariance matrices are defined as $\begin{pmatrix} var(F_1) & cov(F_1, F_2) \\ cov(F_1, F_2) & var(F_2) \end{pmatrix}$.

| Phone | Mean ($\mu$) | Covariance ($\Sigma$) |
|---|---|---|
| /a/ | $(6.1148 \quad 8.8597)$ | $\begin{pmatrix} 0.001128 & -0.00199 \\ -0.00199 & 0.00962 \end{pmatrix}$ |
| /i/ | $(3.1784 \quad 11.2910)$ | $\begin{pmatrix} 0.01522 & -0.00578 \\ -0.00578 & 0.00248 \end{pmatrix}$ |
| /u/ | $(4.3340 \quad 7.0888)$ | $\begin{pmatrix} 0.0104 & 0.01124 \\ 0.01124 & 0.02427 \end{pmatrix}$ |
| /p/ | $(3.3362 \quad 8.0357)$ | $\begin{pmatrix} 0.0011 & -0.0019 \\ -0.0019 & 0.01217 \end{pmatrix}$ |
| /t/ | $(3.2598 \quad 9.6076)$ | $\begin{pmatrix} 0.00127 & -0.00204 \\ -0.00204 & 0.0097 \end{pmatrix}$ |
| /#/ | $(0.0 \quad 0.0)$ | $\begin{pmatrix} 0.001128 & -0.00199 \\ -0.00199 & 0.00962 \end{pmatrix}$ |

presented simulations, it mostly controls information decay speed, that is to say, how fast decoders return to their initial, uniform state in the absence of stimulus. Such decays can be observed in portions of Figure 5 (e.g., probability for syllable "pa" in the first decoder, in late iterations).

3. As in perceptual models, in sensory models, all probability distributions of the form $P(WS^t)$, $P(SyS_i^t)$ and $P(FeS_j^t)$ are defined by discrete uniform probability distributions over their respective domains.
4. $P(I_j^t \mid [FeS_j^t = f])$: for every phone $f$, the probability distribution over spectral contents of the acoustic signals is defined by a multivariate Gaussian distribution $\mathcal{N}(\mu, \Sigma)$ in the space of the first two formants $F_1, F_2$. Their parameters are given Table S1 for all the phones except the transitional phone $/@/$, which has a fixed probability value over the formant domain, arbitrarily set to $10^{-2}$.
5. $P(FeL_j^t \mid [SyS_i^t = s])$: for every syllable $s$, the probability distribution over the phones it has at position $j$ is a Dirac probability distribution, that is to say, a discrete distribution with probability 1 for phone $j$, and probability 0 for other phones. We note:

$$P([FeL_j^t = f] \mid [SyS_i^t = s]) = \begin{cases} 1 & \text{if phone } f \text{ is at position } j \text{ of syllable } s, \\ 0 & \text{otherwise.} \end{cases}$$

6. $P(SyL_i^t \mid [WS^t = w])$: for every word $w$, the probability distribution over the syllables it has at position $i$ is a Dirac probability distribution, that is to say, a discrete distribution with probability 1 for syllable $i$, and probability 0 for other syllables. We note:

$$P([SyL_i^t = s] \mid [WS^t = w]) = \begin{cases} 1 & \text{if syllable } s \text{ is at position } i \text{ of word } w, \\ 0 & \text{otherwise.} \end{cases}$$

7. Perceptual and lexical variables are connected by coherence variables, so that the terms associated, by definition, are specified by:

$$P([\lambda SyPL_i^t = 1] \mid [SyP_i^t = s_p]\ [SyL_i^t = s_l]) = \begin{cases} 1 & \text{if } s_l = s_p \\ 0 & \text{otherwise.} \end{cases}$$

$$P([\lambda FePL_j^t = 1] \mid [FeP_j^t = f_p]\ [FeL_j^t = f_l]) = \begin{cases} 1 & \text{if } f_l = f_p \\ 0 & \text{otherwise.} \end{cases}$$

$P([\lambda WSP^t = 1] \mid [WS^t = w_s]\ [WP^t = w_p])$ is defined in the same manner:

$$P([\lambda WSP^t = 1] \mid [WS^t = w_s]\ [WP^t = w_p]) = \begin{cases} 1 & \text{if } w_s = w_p \\ 0 & \text{otherwise} \end{cases}$$

Technical details about the properties deriving from this definition of coherence variables are found in Section A.5.

8. Sensory and perceptual variables are connected by controlled coherence variables, so that the terms associated, by definition, are specified by:

$$P([\lambda SySP_i^t = 1] \mid [SyS_i^t = s_s]\ [SyP_i^t = s_p]\ [A^t = a_s])$$
$$= \begin{cases} 1 & \text{if } s_s = s_p \text{ and } a_s = 1 \\ 0 & \text{if } s_s \neq s_p \text{ and } a_s = 1 \\ 1/|Sy| & \text{if } a_s = 0 \end{cases}$$

$$P([\lambda FeSP_j^t = 1] \mid [FeS_j^t = f_s]\ [FeP_j^t = f_p]\ [A^t = a_s])$$
$$= \begin{cases} 1 & \text{if } f_s = f_p \text{ and } a_s = 1 \\ 0 & \text{if } f_s \neq f_p \text{ and } a_s = 1 \\ 1/|Fe| & \text{if } a_s = 0. \end{cases}$$

For both expressions, the value of $a_s$ controls the sequential activation of the corresponding decoders. At any given time step, only one syllabic decoder is activated, with $a_s = 1$ for this decoder, and $a_s = 0$ for others. The same applies to phonetic decoders within syllabic decoders. Technical details about the properties deriving from this definition of controlled coherence variables are found in Section A.5.

9. $P(A_{1:15}^t)$ are defined as discrete prior probability distributions, chosen as a function of a decision process applied to the result of an inference over variable $OC^{t-1}$ (see below, Section A.4). If this computation finds that the probability that $OC^{t-1}$ is *True* is larger than a decision threshold $\tau$ (set empirically to 0.4 in the reported experiments), then, for sequencing phone decoders, the current decoder $i$ is closed (by setting the probability $P([A_i^t = 1])$ to 0), and the next decoder $i+1$ is opened (by setting the probability $P([A_{i+1}^t = 1])$ to a small, non-zero value $\alpha_{Fe}$). A similar mechanism is employed for sequencing syllable decoders.

10. $P(OBU^t \mid \Delta L_j^t)$ is defined with a scaled logistic function:

$$P([OBU^t = True] \mid [\Delta L_j^t = \delta_L]) = \begin{cases} 2 \times logistic(uphillC) - 1 & \text{if} \quad \delta_L \geq 0 \\ 0 & \text{else} \end{cases}$$

where *uphillC* is a discrete counter, used to count the number of time steps where energy in the signal envelope was increasing, *logistic* is the logistic function,

$$\mathit{logistic}(x) = \frac{1}{1 + \exp\left(\frac{-(x-\mu)}{s}\right)} \ ,$$

with $\mu$ and $s$ parameters, respectively the mean and a scale parameter proportional to the standard deviation. For all the simulations presented, their values are respectively set to 0 and 1.

11. $P(\Delta L_j^t)$ are probability distributions of the derivatives of the loudness of the stimulus; they are defined as uniform probability distributions (in practice, since values for variables $\Delta L_d^t$ are provided by the stimulus, this choice is arbitrary and without consequence).
12. The term $P(\mathit{Sil}_j^t \mid \mathit{WS}^t)$ is defined by:

$$P(\mathit{Sil}_j^t = 1 \mid \mathit{WS}^t = w_s) = \mathit{logistic}(\mathit{duration}(w_s)) \ ,$$

with *logistic* the same logistic function, with same parameters, as in the $P(\mathit{OBU}^t \mid \Delta L_j^t)$ term, and $\mathit{duration}(w_s)$ a function that provides, for each known word, its duration in time steps according to the lexicon (see Table 2).

13. The term $P(\mathit{OTD}^t \mid \mathit{WS}^t)$ is defined by:

$$P([\mathit{OTD}^t = \mathit{True}] \mid [\mathit{WS}^t = w_s]) = \sum_{s \in w_s} \mathcal{N}(\mu = \mathit{TimeOnset}(s, w_s), \sigma^2)$$

where $\mathcal{N}$ is the Normal probability density function, and $\mathit{TimeOnset}(s, w_s)$ is a function providing the time-instant at which the syllabic onset of syllable $s$ in word $w_s$ is expected. For all the presented simulations, the variance $\sigma^2$, controlling the dispersion of the temporal windows in which onsets are expected, is set to an arbitrary value 10. One "normal kernel" is associated to predict each syllabic onset in a word, and they are summed to form the probability of onsets over all time steps. Figure S1 shows an illustration of the resulting probability profile, over all time steps, for the word *"pata"*. Since it is a bi-syllabic word, there are two normal kernels, around time steps 0 and 150.

14. The $P(\mathit{OREF}^t \mid {A_{1:15}}^t)$ term implements the refractory period, that is to say, it sets the probability to have another onset event to 0 for the next 50 time steps after the last detected onset. Technically, it is a conditional Dirac probability distribution, so that $P([\mathit{OREF}^t = \mathit{True}] \mid {A_{1:15}}^t)$ is 0 if the last time-instant at which ${A_{1:15}}^t$ was *True* is not yet "old enough" (this relies on an internal counter to describe this "memory", which is technically "outside" of the probabilistic description of the model, for simplicity).
15. The final term, $P(\mathit{OC}^t \mid \mathit{OTD}^t \ \mathit{OBU}^t \ \mathit{OREF}^t)$, defines the fusion operator. In the *AND* variant of the fusion operator, we define

$$\begin{aligned} & P([\mathit{OC}^t = T] \mid [\mathit{OTD}^t = T] \ [\mathit{OBU}^t = T] \ [\mathit{OREF}^t = T]) \\ = \ & P([\mathit{OTD}^t = T]) \times P([\mathit{OBU}^t = T]) \times P([\mathit{OREF}^t = T]) \ , \end{aligned}$$

with $T$ for the *True* Boolean value. This implements a combination in which the probability that there is an onset is the product of probability of the three "temporal submodels" (the top-down prediction, sensory detection, and refractory period). As a consequence of this product, the probability value can

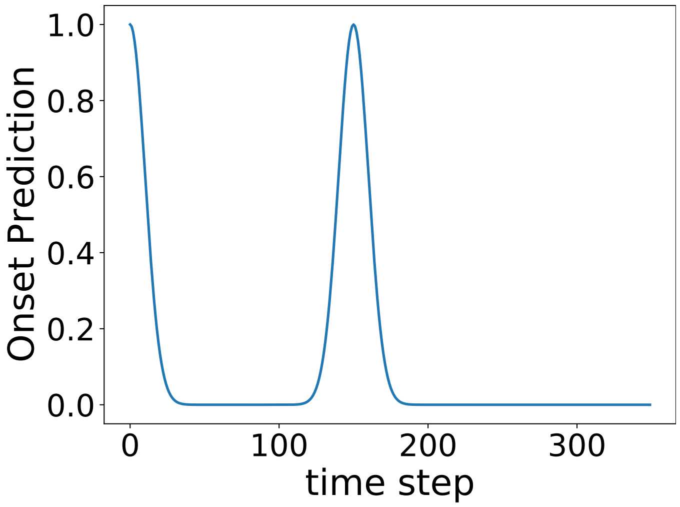


**Figure S1.** Top-down temporal prediction of syllabic onsets for the word *"pata"*, in the term $P([\mathit{OTD}^t = \mathit{True}] \mid [\mathit{WS}^t = \mathit{pata}])$. On the $x$-axis, the simulated time steps, and on the $y$-axis, the probability of predicted onsets at different time steps.

be close to one only when the three components agree and also provide high probability that there is an onset; this explains why we denote this an "*AND*" combination. To define the *OR* operator, we apply De Morgan's law, $A \vee B = \overline{\overline{A} \wedge \overline{B}}$, and define:

$$\begin{aligned} & P([\mathit{OC}^t = T] \mid [\mathit{OTD}^t = T]\ [\mathit{OBU}^t = T]\ [\mathit{OREF}^t = T]) \\ = \ & \big(1 - (1 - P([\mathit{OTD}^t = T])) \times (1 - P([\mathit{OBU}^t = T]))\big) \times P([\mathit{OREF}^t = T])\ . \end{aligned}$$

Therefore, notice that, since the *AND* or *OR* fusion between bottom-up and top-down event detection is applied before the refractory process occurs, the *AND* opertaor could be construed as a probabilistic implementation of "*OTD AND OBU AND OREF*", while the *OR* operator could be noted as a probabilistic "(*OTD OR OBU*) *AND OREF*".

## A.4 Inference for simulating word recognition

Here, we detail how word recognition and onset detection are computed in the model. Both correspond to probabilistic computations, computed in an online manner, thanks to recursive solutions provided by Bayesian inference. Both word recognition and onset detection are thus computed at each time step: word recognition proceeds assuming the states of the phone and syllable decoders as given, and onset detection, informed by word recognition, proceeds to compute the states of phones and syllables decoders for the next time step. In other words, model simulation proceeds in an iterative manner, as only probability distributions at time $t$ are needed to compute probability distributions at time $t + 1$ (even though, for visualization purposes, we also memorize the whole history of probability distributions, this is not required for simulations).

Consider first word decoding. Formally, word decoding relies on phone decoding and syllable decoding. To simulate these, we compute the probability distributions over the perceived phones *FeP*, syllables *SyP* and word *WP*, at each time step, assuming that the stimulus and states of each phone and syllable decoders (i.e., whether they are active or not) are given. To differentiate these three computations, we use the coherence variables to limit the propagation of information extracted from the stimulus into the model.

Consider first phone decoding. We are thus interested in computing, $\forall j, t$, $\mathcal{Q}Fe_j^t = P(FeP_j^t \mid I_j^{0:t}[\lambda FeSP_j^{0:t} = 1])$. Applying Bayesian inference in the model yields:

$$\mathcal{Q}Fe_j^t \propto \left( \begin{array}{l} [\alpha_{Fe} P(I_j^t \mid FeS_j^t) + (1 - \alpha_{Fe})\mathcal{U}_{Fe}] \\ \times \sum_{FeP_j^{t-1}} P(FeP_j^t \mid FeP_j^{t-1})\ \mathcal{Q}Fe_j^{t-1} \end{array} \right), \tag{S1}$$

where $\alpha_{Fe}$ is either equal to a constant (set to $10^{-1}$ for the simulations) when the corresponding phonetic decoder is activated, or 0 otherwise, and $\mathcal{U}_{Fe}$ is the uniform probability value over the phone space.

In a similar manner, for syllable decoding, we compute $\mathcal{Q}Sy_i^t = P(SyP_i^t \mid I_J^{0:t}[\lambda SySP_i^{0:t} = 1]\ [\lambda FeSP_J^{0:t} = 1])$ (with $J$ denoting the set of subscripts from $4(i-1)+1$ to $4i$). Applying Bayesian inference yields:

$$\mathcal{Q}Sy_i^t \propto \left( \begin{array}{l} \prod_{j=4(i-1)+1}^{4i} [\alpha_{Sy} \langle P(FeL_j^t \mid SyS_i^t), \mathcal{Q}Fe_j^t \rangle + (1 - \alpha_{Sy})\mathcal{U}_{Sy}] \\ \times \sum_{SyP_i^{t-1}} P(SyP_i^t \mid SyP_i^{t-1})\ \mathcal{Q}Sy_i^{t-1} \end{array} \right), \tag{S2}$$

where $\alpha_{Sy}$ is either equal to a constant (set to $10^{-2}$ for the simulations) when the corresponding syllabic decoder is activated, or 0 otherwise, and $\mathcal{U}_{Sy}$ is the uniform probability value over the syllable space.

Finally, for word decoding, we compute $\mathcal{Q}W^t = P(WP^t \mid I_{1:12}^{0:t}\ Sil_{1:12}^{0:t}\ [\lambda WSP^{0:t} = 1]\ [\lambda SySP_{1:3}^{0:t} = 1]\ [\lambda FeSP_{1:12}^{0:t} = 1]))$. Applying Bayesian inference yields:

$$\mathcal{Q}W^t \propto \left( \begin{array}{l} P(Sil \mid WS^t) \\ \times \prod_{i=1}^{3} [\alpha_W \langle P(SyL_i^t \mid WS^t), \mathcal{Q}Sy_i^t \rangle + (1 - \alpha_W)\mathcal{U}_W] \\ \times \sum_{WP^{t-1}} P(WP^t \mid WP^{t-1})\ \mathcal{Q}W^{t-1} \end{array} \right), \tag{S3}$$

where $\alpha_W$ is equal to a constant (set to $10^{-2}$ for the simulations) and $\mathcal{U}_W$ is the uniform probability value over the word space.

We note that these inferences are approximate inference. First, we do not take into account the feedback loops required by the complete, loopy dependency structure of the probabilistic model (due to the different Markov chains in parallel). Indeed, even though it is represented as a tree in Figure 1, the dependency structure of the decoding module contains variables with self-loops: in other words, the decoding module is a set of Markov chains, one over each such variable, interacting at each time step through the dependency structure shown in Figure 1. In such a hierarchical dynamic model, exact Bayesian inference would require sophisticated techniques, and even approximate Bayesian inference would require feed-forward and feedback information propagation until numerical convergence. Here, we proceed in a single-pass forward inference, as a first, rough approximation. (However, we note that introducing a feedback pass in a model with a similar architecture (Phénix, 2018) enabled contextual effects to appear in decoding; in our case, this would provide context effects of word-recognition for syllable-decoding, and of syllable-recognition for phone-decoding. These effects are outside of the scope of the current model.) Second, we also do not consider the information propagation to the temporal module, and consider inference in the decoding portion of the model as independent (it "receives", from the temporal module, only onset events, and not probability distributions over onsets; in other words the temporal module is seen, by the decoding module, as an external, independent sensor providing the states (open or closed) of phone and syllable decoders).

Consider, second, onset detection. At each time step, once word decoding is computed, we then compute, in the temporal module, onset detection, to update the states of phone and syllable decoders for the next, upcoming time step.Inference for the bottom-up, sensory detection of onsets simply proceeds by referring

to the $P(OBU^t \mid \Delta L_j^t)$ term. On the other hand, for the top-down, prediction of onsets, we compute the probability:

$$P([OTD^t = True]) = \sum_{ws^t} P([OTD^t = True] \mid [WS^t = ws^t]) \; QW^t \; .$$

In other words, we compute the probability that there would be an onset, according to the lexical models of all words, simultaneously, but weighed according to the current probability distribution over words as computed by word recognition.

Computations of the probabilities of onsets in the refractory model, and in the fusion model, simply proceed by applying the corresponding definitions of their probabilistic terms. Considering, for instance, the *AND* fusion operator, we thus compute:

$$\begin{aligned} & P([OC^t = True] \mid \Delta L_{1:12}^t \; Sil_{1:12}^t \; I_{1:12}^t [\Lambda = 1]) \\ = & \left( \sum_{ws^t} P([OTD^t = True] \mid [WS^t = ws^t]) \; QW^t \right) \\ & \times P([OBU^t = T] \mid \Delta L_j^t) \times P([OREF^t = T]) \; . \end{aligned}$$

with $j$ the index of the current active phone decoder, and $\Lambda$ representing the set of all coherence variables of the decoding module.

The final step of onset detection is to apply the decision process on the computed probability distribution: when the probability that $[OC^t = True]$ is above a threshold, an onset is considered to be detected, which updates the states of phone and syllable decoders. Technically, this is done by changing the prior distributions over $P(A_{1:15}^{t+1})$ (see Section A.3). This final step is not properly a "probabilistic dependency" in the model; that is why it is represented as a dotted arrow in Figure 1.

## A.5 Using coherence and controlled coherence variables for controlling decoder input

Here, we detail how coherence variables and controlled coherence variables, can be used to control, in the model, when decoders are fed with sensory input. To do so, we consider a small portion of the model around the first phone decoder (without loss of generality, as this would apply to other phone decoders and to syllable decoders, as well).

Consider thus the first phone decoder. To recall, it is essentially a temporal model over variables $FeP_1^{0:T}$, defined by a dynamic model $P(FeP_1^t \mid FeP_1^{t-1})$. To simplify, we consider it provides, at time $t$, a distribution over perceived phones, noted here $P(FeP_1^t)$. This temporal model is fed sensory information from the input; this involves an inversion of the term $P(I_j^t \mid FeS_1^t)$. To simplify, we consider it provides, at time $t$, a distribution over sensed phones, noted here $P(FeS_1^t)$. We connect these distributions with a simple coherence variable $\lambda FeSP_1^t$. Therefore, we consider the model $P(FeP_1^t \; FeS_1^t \; \lambda FeSP_1^t)$, defined by:

$$P(FeP_1^t \; FeS_1^t \; \lambda FeSP_1^t) = P(FeP_1^t) P(FeS_1^t) P(\lambda FeSP_1^t \mid FeP_1^t \; FeS_1^t) \; ,$$

with the term $P(\lambda FeSP_1^t \mid FeP_1^t \; FeS_1^t)$ defined by:

$$P([\lambda FeSP_1^t = 1] \mid [FeP_1^t = f_p] \; [FeS_1^t = f_s]) \; = \; \begin{cases} 1 & \text{if } f_p = f_S \\ 0 & \text{otherwise.} \end{cases}$$

We now demonstrate that, in this simplified portion of the model, coherence variable $\lambda FeSP_1^t$ can be employed as a "Bayesian switch", that is, we can choose during inference, whether information propagates from sensory information about phones to the phone decoder, or not. These demonstrations are adapted from other texts about coherence variables as Bayesian switches (Bessière et al., 2013; Gilet et al., 2011).

First, consider computing $P(FeP_1^t)$ in the model as defined above. The result of Bayesian inference can be shown to be equal to $P(FeP_1^t)$, since it appears as is in the decomposition of the joint probability distribution. By assumption, $P(FeP_1^t)$ is thus independent of the sensory distribution $P(FeS_1^t)$. Here, the coherence variable is unspecified, and this can be interpreted as "opening" the Bayesian switch. In other words, whatever information is in $P(FeS_1^t)$, it does not affect $P(FeP_1^t)$.

Second, consider computing $P([FeP_1^t = f_p] \mid [\lambda FeSP_1^t = 1])$:

$$
\begin{aligned}
& P([FeP_1^t = f_p] \mid [\lambda FeSP_1^t = 1]) \\
& \quad \propto \sum_{FeS_1^t} P([\lambda FeSP_1^t = 1]\ [FeP_1^t = f_p]\ FeS_1^t) \\
& \quad \propto \sum_{FeS_1^t} P([FeP_1^t = f_p]) P(FeS_1^t) P([\lambda FeSP_1^t = 1] \mid [FeP_1^t = f_p]\ FeS_1^t) \; .
\end{aligned}
$$

In the summation over variable $FeS_1^t$, the term $P([\lambda FeSP_1^t = 1] \mid [FeP_1^t = f_p]\ [FeS_1^t = f_s])$ is always 0 except when $f_p = f_s$, so that the summation can be collapsed:

$$
\begin{aligned}
& P([FeP_1^t = f_p] \mid [\lambda FeSP_1^t = 1]) \\
& \quad \propto P([FeP_1^t = f_p]) P([FeS_1^t = f_p]) P([\lambda FeSP_1^t = 1] \mid [FeP_1^t = f_p]\ [FeS_1^t = f_p]) \\
& \quad \propto P([FeP_1^t = f_p]) P([FeS_1^t = f_p]) \; .
\end{aligned}
$$

Therefore, $P([FeP_1^t = f_p] \mid [\lambda FeSP_1^t = 1])$ is not independent of $P(FeS_1^t)$. In other words, setting the coherence variable $\lambda FeSP_1^t$ to value 1 "closes" the Bayesian switch: contrary to the previous case, here, sensory information in $P(FeS_1^t)$ is combined with the distribution $P(FeP_1^t)$, and the combination operator is, mathematically, a product of the two probability distributions.

A technical precision can be raised here. Even though the definition above could suggest that a coherence variable "forces" the variables that it connects to be equal, this is not so in effect. Indeed, it is true that a coherence variable imposes equality during inference, but this merely allows to "collapse summations" over the adjacent variables. This results in mathematical forms with products of probability distributions, such as, in our example, $P([FeP_1^t = f_p])P([FeS_1^t = f_p])$. In this expression, whereas it is true that the product is performed "assuming that variables have the same value", this does not imply any constraints on probability distributions $P(FeP_1^t)$ and $P(FeS_1^t)$. Indeed, these can "mostly agree", with their probability masses concentrated on the same values in their domain, or these can be "widely in conflict", with their probability masses on different portions of their domain, or any other situation in between. The mathematical machinery of coherence variables is agnostic to this, and always results in a "fusion model" that is a product of distribution.

We now consider a slightly more complex example, in which coherence variable $\lambda FeSP_1^t$ would get controlled by an additional variable, $A_1^t$. The decomposition of the joint probability distribution

$P(\mathit{FeP}_1^t\ \mathit{FeS}_1^t\ \lambda\mathit{FeSP}_1^t\ A_1^t)$ would become:

$$P(\mathit{FeP}_1^t\ \mathit{FeS}_1^t\ \lambda\mathit{FeSP}_1^t\ A_1^t) = P(\mathit{FeP}_1^t)P(\mathit{FeS}_1^t)P(A_1^t)P(\lambda\mathit{FeSP}_1^t \mid \mathit{FeP}_1^t\ \mathit{FeS}_1^t\ A_1^t)\ ,$$

with the term $P(\lambda\mathit{FeSP}_1^t \mid \mathit{FeP}_1^t\ \mathit{FeS}_1^t\ A_1^t)$ defined by:

$$\begin{aligned} & P([\lambda\mathit{FeSP}_1^t = 1] \mid [\mathit{FeS}_j^t = f_s]\ [\mathit{FeP}_j^t = f_p]\ [A_1^t = a]) \\ = & \begin{cases} 1 & \text{if } f_s = f_p \text{ and } a = 1 \\ 0 & \text{if } f_s \neq f_p \text{ and } a = 1 \\ 1/|\mathit{Fe}| & \text{if } a = 0. \end{cases} \end{aligned}$$

This is the same definition for this term as in the full model described above.

We now demonstrate that, with this definition of the model, the controlled coherence variable allows gradual control of information propagation in the model (Phénix, 2018). We consider, as above, computing:

$$\begin{aligned} & P([\mathit{FeP}_1^t = f_p] \mid [\lambda\mathit{FeSP}_1^t = 1]) \\ \propto\ & \sum_{\mathit{FeS}_1^t, A^t} P([\lambda\mathit{FeSP}_1^t = 1]\ [\mathit{FeP}_1^t = f_p]\ \mathit{FeS}_1^t\ A_1^t) \\ \propto\ & \sum_{\mathit{FeS}_1^t, A_1^t} P([\mathit{FeP}_1^t = f_p])P(\mathit{FeS}_1^t)P(A_1^t)P([\lambda\mathit{FeSP}_1^t = 1] \mid [\mathit{FeP}_1^t = f_p]\ \mathit{FeS}_1^t\ A_1^t)\ . \\ \propto\ & P([\mathit{FeP}_1^t = f_p]) \left( \begin{array}{l} P([A_1^t = 1]) \sum_{\mathit{FeS}_1^t} P(\mathit{FeS}_1^t)P([\lambda\mathit{FeSP}_1^t = 1] \mid [\mathit{FeP}_1^t = f_p]\ \mathit{FeS}_1^t\ [A_1^t = 1]) \\ +P([A_1^t = 0]) \sum_{\mathit{FeS}_1^t} P(\mathit{FeS}_1^t)P([\lambda\mathit{FeSP}_1^t = 1] \mid [\mathit{FeP}_1^t = f_p]\ \mathit{FeS}_1^t\ [A_1^t = 0]) \end{array} \right) \\ \propto\ & P([\mathit{FeP}_1^t = f_p]) \left( P([A_1^t = 1])P([\mathit{FeS}_1^t = f_p]) + P([A_1^t = 0]) \frac{1}{|\mathit{Fe}|} \right)\ . \end{aligned}$$

As in Equation (S1), we note $P([A_1^t = 1]) = \alpha_{Fe}$, and rewrite this last result:

$$\begin{aligned} & P([\mathit{FeP}_1^t = f_p] \mid [\lambda\mathit{FeSP}_1^t = 1]) \\ \propto\ & P([\mathit{FeP}_1^t = f_p]) \left( \alpha_{Fe} P([\mathit{FeS}_1^t = f_p]) + (1 - \alpha_{Fe}) \frac{1}{|\mathit{Fe}|} \right)\ . \end{aligned}$$

To interpret this result, consider two extreme cases. First, when $\alpha_{Fe} = 1$, this result is identical to the simple case, and the two distributions over $\mathit{FeP}_1^t$ and $\mathit{FeS}_1^t$ are multiplied together: therefore, $\alpha_{Fe} = 1$ would correspond to fully closing the Bayesian switch. Second, when $\alpha_{Fe} = 0$, the distribution over $\mathit{FeP}_1^t$ is multiplied with a uniform distribution, which leaves it unchanged (the uniform distribution is the neutral element for the product of probability distributions), so that the distribution over $\mathit{FeP}_1^t$ is independent of the one over $\mathit{FeS}_1^t$: therefore, $\alpha_{Fe} = 0$ would correspond to fully opening the Bayesian switch. In the general case however, $\alpha_{Fe}$ is neither 0 nor 1, which allows mixing the above two computations: the Bayesian switch is simultaneously "open" and "closed", in amounts controlled by $\alpha_{Fe}$. This allows controlling, in a gradual manner, the amount of information propagated from the sensory distribution to the perceptual model. Therefore, the controlled coherence variable structure can be interpreted, not as a Bayesian switch, but as a Bayesian potentiometer (to pursue the electric analogy; a potentiometer allows gradual control of electric resistance, whereas a switch controls it in an all-or-nothing manner).

In the context of our model of speech decoding, variables $A^t_{1:15}$ are in charge of controlling which phone or syllable decoder receives sensory information; their distributions pilot all links between decoders and their respective sensory inputs. When the probability that the control variable $A^t_i$ is True is 0, the corresponding decoder is not yet activated or already terminated; on the other hand, when the probability that the control variable $A^t_i$ is True has a small, non-zero value, the corresponding decoder is currently activated, so that a small amount of sensory information is fed into the perceptual model.

## REFERENCES

Bessière, P., Laugier, C., and Siegwart, R. (eds.) (2008). *Probabilistic Reasoning and Decision Making in Sensory-Motor Systems*, vol. 46 of *Springer Tracts in Advanced Robotics* (Berlin: Springer)

Bessière, P., Mazer, E., Ahuactzin, J. M., and Mekhnacha, K. (2013). *Bayesian Programming* (Boca Raton, Florida: CRC Press)

Diard, J. (2015). *Bayesian Algorithmic Modeling in Cognitive Science*. Habilitation à diriger des recherches (HDR), Université Grenoble Alpes

Ghitza, O. (2011). Linking speech perception and neurophysiology: speech decoding guided by cascaded oscillators locked to the input rhythm. *Frontiers in Psychology* 2, 130

Gilet, E., Diard, J., and Bessière, P. (2011). Bayesian action-perception computational model: Interaction of production and recognition of cursive letters. *PLoS ONE* 6, e20387

Ginestet, E., Phénix, T., Diard, J., and Valdois, S. (2019). Modeling the length effect for words in lexical decision: The role of visual attention. *Vision Research* 159, 10–20

Lebeltel, O., Bessière, P., Diard, J., and Mazer, E. (2004). Bayesian robot programming. *Autonomous Robots* 16, 49–79

McClelland, J. L. and Elman, J. L. (1986). The TRACE model of speech perception. *Cognitive Psychology* 18, 1–86

Phénix, T. (2018). *Modélisation bayésienne algorithmique de la reconnaissance visuelle de mots et de l'attention visuelle*. Ph.D. thesis, Univ. Grenoble Alpes
